# Causal discovery and inference in COVID-19: A multiscale longitudinal analysis using mixtures of DAGs

**DOI:** 10.1371/journal.pone.0355553

**Published:** 2026-10-30

**Authors:** Kosar Abdollahi, Zahra Rezaei Ghahroodi, Marjan Mansourian

**Affiliations:** 1 Department of Statistics, School of Mathematics, Statistics and Computer Science, College of Science, University of Tehran, Tehran, Iran; 2 Department of Epidemiology and Biostatistics, School of Health, Isfahan University of Medical Sciences, Isfahan, Iran; South China Normal University, CHINA

## Abstract

Real-world longitudinal clinical datasets present major challenges for causal discovery due to mixed variable types, temporal dependencies, treatment-selection mechanisms, missingness, and patient heterogeneity. We extended the Causal Inference over Mixtures (CIM) framework to accommodate mixed-type longitudinal data and applied it to 1,937 hospitalized patients from the Khorshid COVID Cohort. The analysis integrated admission characteristics, symptom trajectories, in-hospital treatments, laboratory measures, and short-term outcomes up to 12 weeks after discharge using mixed conditional-independence testing, bootstrap stability selection, graph-based interpretation, and follow-up outcome assessment. The recovered ancestral structures revealed clinically coherent symptom clusters at admission and persistent associations among several post-discharge symptoms, including cough, fatigue/weakness, shortness of breath, weight loss, and loss of smell. Treatment-related associations were interpreted cautiously because cumulative treatment exposure may reflect disease severity, hospitalization duration, physician decision-making, and other selection mechanisms. In particular, the observed associations involving ceftriaxone and interferon-β should be regarded as exploratory and require independent validation. Simulation studies demonstrated that CIM consistently outperformed PC, FCI, RFCI, and CCI, achieving the highest recovery accuracy and balanced accuracy while maintaining low false-positive rates and competitive computational efficiency, even in the presence of latent confounding and selection bias. These results support the feasibility and utility of the proposed framework for identifying robust structural patterns in complex longitudinal clinical data. Given the observational nature of the study, the single-cohort design, and the absence of external replication, the findings should be viewed primarily as hypothesis-generating rather than confirmatory evidence of causal effects.

## 1 Introduction

In late 2019, a cluster of patients began to experience the symptoms of an atypical pneumonia-like illness that did not respond well to standard treatments. This disease, known as coronavirus disease 2019 (COVID-19), rapidly spread across the globe and created significant challenges in healthcare, the economy, and daily life [1]. In light of these challenges, scientific investigation became essential. COVID-19 research contains multiple dimensions, including epidemic modeling, diagnosis and identification, impact assessment, simulation studies, and the analysis of resurgence and mutation patterns [2]. To address these research dimensions, a broad range of methods has been applied, including traditional statistical techniques, compartmental models, machine learning, deep learning, and simulation-based approaches.

Although these methods have provided powerful tools for prediction and classification, they primarily capture statistical associations rather than causal relationships. This distinction is particularly important in healthcare: association-based predictions may support risk assessment, but understanding why an outcome occurs requires identifying the underlying causal mechanisms, which is essential for effective treatment. For example, a model may predict that patients prescribed a certain medication are more likely to experience a specific symptom; however, this does not necessarily imply a causal effect of the medication. Without clearly distinguishing between correlation and causation and understanding the underlying mechanisms, data-driven decisions may be misleading.

While most existing approaches predominantly rely on supervised learning, relatively few have explored unsupervised or structure-learning techniques–and even fewer have sought to uncover underlying causal mechanisms [3]. Although some unsupervised learning methods can capture structures within the data, they remain insufficient for addressing the types of causal questions that are central to clinical decision-making such as whether a treatment genuinely improves outcomes, or whether a symptom reflects a deeper pathological process requiring targeted intervention. Consequently, the ability to infer causal relationships from data is essential for enabling more precise and effective clinical interventions.

In many data-driven disciplines, particularly healthcare and epidemiology, understanding causal relationships is essential for reliable decision-making. However, establishing causality is a complex process; as causal relationships cannot be read off from the data but have to be inferred [4]. While Randomized Controlled Trials (RCTs) are widely regarded as the gold standard for establishing causal effects under controlled experimental conditions, they are often infeasible, costly, or ethically constrained, particularly in large-scale or emergency settings such as global pandemics [5]. Therefore, developing frameworks that can infer causal relationships from observational data has become increasingly important for advancing scientific understanding [6].

The field of causal discovery has emerged to address this challenge. It complements the more familiar task of causal inference, which estimates the strength of causal effects given a known or assumed structure. In contrast, causal discovery aims to learn the underlying causal structure directly from observational data under a set of principles and assumptions. Causal discovery algorithms are typically classified into two main categories: score-based and constraint-based methods. Constraint-based approaches infer the underlying causal graph by leveraging conditional independence relationships derived from the data, whereas score-based methods explore the space of candidate structures by optimizing a model selection criterion. This study focuses on constraint-based approaches.

These methods rely on several key assumptions. Two core principles are the causal Markov condition and the faithfulness assumption, which together imply a correspondence between the (conditional) independencies observed in the probability distribution and the causal connectivity relations in the graph. Causal connectivity is formally defined in terms of d-separation and d-connection [4]. In addition, most approaches assume causal sufficiency, meaning that there are no unmeasured common causes of the observed variables, and acyclicity, whereby the system can be represented as a directed acyclic graph (DAG) without feedback loops. Another common assumption is the absence of selection bias, which may arise when the dataset is conditioned on variables affecting inclusion (e.g., hospitalization), potentially distorting observed associations. A wide range of causal discovery methods has been developed under different assumptions about the data-generating process; for a comprehensive overview, see [7].

Fig 1 illustrates how constraint-based causal discovery methods have evolved over time. However, most of these are designed for cross-sectional data and assume a single causal structure for the entire dataset. These assumptions are limiting when dealing with clinical datasets, which often involve heterogeneous populations, longitudinal measurements, selection mechanisms, and latent variables.

**Fig 1:**
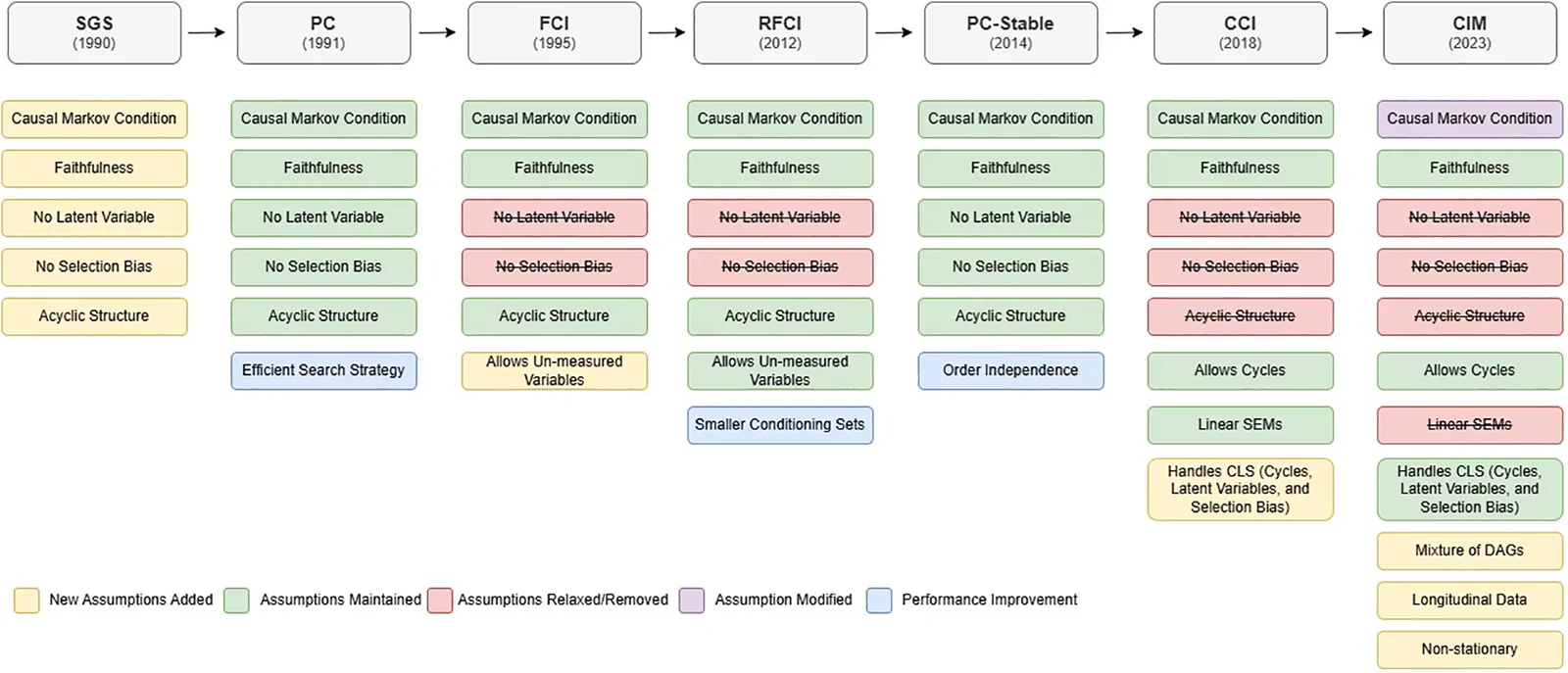
Evolution of Constraint-Based Causal Discovery Algorithms. Each row represents a key assumption or algorithmic feature. Color coding indicates their status over time: green for unchanged assumptions, red for relaxed or removed ones, blue for performance improvements (e.g., increased efficiency), yellow for newly introduced assumptions, and purple for modified assumptions.

Most existing research on causal discovery in longitudinal studies has focused on time series analysis. Granger-Causality (GC) [8] remains a foundational approach, inspiring methods such as PCMCI [9] and TsFCI [10]. Based on GC, Biazoli et al. [11] developed a method for analyzing large-scale cohort studies with two time points, where potential causes are measured at baseline and outcomes are evaluated during follow-up. Longitudinal LiNGAM [12], which extends the LiNGAM model to longitudinal data by analyzing paired samples over time under assumptions of linearity, non-Gaussian noise, and acyclicity. Saeed et al. [13] showed that the FCI algorithm, can manage non-stationary conditions, provided that a specific variable ordering assumption is maintained over time. This assumption implies that the causal relationships among variables must follow a consistent sequence throughout different time points. However, this ordering can be easily violated in scenarios where the underlying graphical structure shifts or contains cycles. GLACIAL (Granger and Learning-based Causality Analysis) [14] adapts GC to the longitudinal setting by treating subject trajectories as independent samples and using a shared neural forecasting model with dropout and interpolation to capture nonlinear causal dynamics, while managing sparse sampling and missing data. Jin et al. [15] propose a framework using directed cyclic graphs and instrumental variables to capture both time-lagged and instantaneous effects.

A recent and versatile framework, known as the Mixture of Directed Acyclic Graphs (DAGs), was introduced by Strobl [16] to address several limitations of earlier methods. This framework models real-world causal processes as mixture of DAGs, where each sample follows a joint distribution that factorizes according to a DAG, although the underlying structure may vary across samples. Based on this idea, the Causal Inference over Mixtures (CIM) algorithm was developed to enable causal discovery from longitudinal data under more flexible assumptions. The performance of CIM has been evaluated in prior work [16], showing favorable results compared to existing methods, particularly when the data-generating process involves mixture of causal structures. CIM extends earlier approaches by accommodating arbitrary variable ordering, non-linearity, cycles, non-stationarity, non-parametric distributions, dynamic causal relationships, latent confounding, and selection bias.

However, the current formulation of CIM is limited to continuous variables and has been primarily evaluated on synthetic datasets, thereby restricting its applicability to real-world clinical settings. Extending the framework to accommodate mixed-type data commonly encountered in clinical practice is therefore a necessary step.

Beyond methodological considerations, the existing research on COVID-19 remains limited in scope. Many studies have focused on the early stages of infection — diagnosis, risk prediction, or epidemiological modeling — and rely on statistical associations [17,18]. In addition, many existing studies isolate either diagnosis or treatment, without addressing how the two interact over time [19]. Furthermore, clinical outcome studies frequently model endpoints such as mortality or ICU admission independently, often requiring separate models or employing complex multivariate techniques such as copulas.

Only a limited number of studies have applied graph-based methods to analyze COVID-19 datasets. One example is Zhang et al. [20], who constructed multifactorial causal models to examine the nephrotoxic effects of remdesivir (RDV) in COVID-19–associated acute kidney injury (AKI). Their approach leveraged constraint-based algorithms (FCI and GFCI) and incorporated expert priors, but assumed a single static DAG and did not fully address issues like latent confounding or selection bias. Table 1 provides a broader comparison of this and other recent studies, highlighting key methodological differences, including support for mixed-type variables [21–23] and longitudinal data [24], the structure learning approach, and assumptions about underlying graph.

**Table 1:** Comparison of the proposed method with prior graph-based methods on COVID-19.

Study & Year	Applied to mixed-type data	Longitudinal	Structure Learning Method	Quantifying Effects	Assumptions
**Our Study**	✓	✓	CIM algorithm	✓	Faithfulness; mixture of DAGs; allows cycles
**Zhang et al., 2023** *EHR nephrotoxicity of Remdesivir*	✓	✓	FCI and GFCI algorithms	✓	Faithfulness; single DAG; expert prior knowledge; confounding not interpreted causally; selection bias acknowledged but unhandled; acyclicity enforced
**Ferrari et al., 2022** *Causal learning framework for COVID-19 clinical data*	✓	X	FCI algorithm	✓	Faithfulness; single DAG; expert priors; latent confounding handled; selection bias acknowledged but unhandled; acyclicity enforced
**Strobl, 2023** *Causal discovery with a mixture of DAGs*	X	✓	CIM algorithm	X	Faithfulness; mixture of DAGs; allows cycles
**Constantinou et al., 2023** *Open problems in causal structure learning: A case study of COVID-19 in the UK*	✓	X	29 algorithms benchmarked	X	Faithfulness; single DAG; acyclicity enforced
**Mastakouri & Schölkopf, 2020** *Causal analysis of Covid-19 spread in Germany*	X	✓ (time series)	Modified SyPI algorithm	X	Faithfulness; single DAG; confounding handled; selection bias not discussed; acyclicity enforced
**Steiger et al., 2021** *Causal graph analysis of COVID-19 observational data in German districts reveals effects of determining factors on reported case numbers*	✓	✓ (time series)	Manual (literature-based)	✓	Faithfulness; single DAG; confounding handled; acyclicity enforced

Effectively analyzing complex clinical conditions such as COVID-19 requires methods capable of representing longitudinal structure, mixed variable types, patient heterogeneity, latent confounding, and selection mechanisms. Building on the Causal Inference over Mixtures framework, this study extends the methodology to accommodate mixed-type clinical variables and applies it to a real-world longitudinal COVID-19 cohort. The main contribution is methodological and exploratory, focusing on the evaluation of whether a mixture of DAG framework can recover clinically coherent structures from routinely collected longitudinal hospital data and generate graph-informed hypotheses about symptom persistence, treatment allocation, laboratory markers, and clinical outcomes. This study is not intended to provide definitive causal evidence regarding treatment efficacy or mechanisms underlying long-COVID. Instead, it evaluates the feasibility and interpretability of an extended causal discovery framework in an early-pandemic clinical cohort while explicitly acknowledging the constraints imposed by observational data, short follow-up duration, evolving treatment protocols, and the absence of external validation.

Accordingly, the objectives of this study were threefold: first, to adapt the CIM framework for mixed-type longitudinal clinical data; second, to apply the extended framework to the KCC dataset to identify stable graph structures linking admission characteristics, symptoms, treatments, laboratory findings, and short-term outcomes; and third, to use graph-derived hypotheses to guide cautious follow-up analyses of selected treatment–outcome and symptom-persistence relationships. Given that follow-up data were limited to 12 weeks after discharge, the observed symptom trajectories are interpreted as short-term post-acute outcomes rather than definitive long-COVID.

The remainder of this paper is organized as follows. **Materials and Methods** section describes the KCC dataset, outlines the structure of the longitudinal data, and presents the methodological framework, including preprocessing steps, handling of mixed-type data, and the causal discovery approach used in this study. The **Results** section reports the main findings from our analyses. The **Simulation Study** section presents the simulation framework, including the experimental setup and the synthetic data generation process, to evaluate the performance of the proposed method under mixed-type longitudinal settings. The **Discussion** section interprets the results, highlights their clinical implications, and compares them with existing literature. Finally, The **Conclusion** section summarizes the main findings and outlines directions for future work.

## 2 Materials and methods

This section provides an overview and exploratory analysis of the KCC dataset, followed by a detailed description of our methodological framework based on the Mixture of Directed Acyclic Graphs (DAGs), adapted for longitudinal and mixed-type clinical data.

### 2.1 Dataset description

This study uses data from the KCC, a one-year hospital-based prospective surveillance study conducted in the Eastern Mediterranean region [25]. The KCC study collected data from patients admitted for COVID-19 from 01/02/2020–28/02/2021 at Khorshid Hospital in Isfahan, Iran, a major outbreak zone that served as the primary referral hospital for adult COVID-19 patients in the region, receiving approximately 50% of the total COVID-19 cases in Isfahan; the data were accessed for research purposes on 18/03/2023.

The study population consisted of 1998 COVID-19 patients. Diagnosis was based on either a positive polymerase chain reaction (PCR) test or a high-resolution CT (HRCT) scan compatible with COVID-19, following Chinese COVID-19 diagnosis and treatment guidelines and WHO provisional advice.

Data collection was conducted in two distinct phases. The first phase, the Hospital Admission Phase, included information collected during hospitalization until discharge or death. The second phase, the Follow-up Phase, included post-discharge assessments at weeks 1, 4, and 12. Telephone follow-ups at weeks 1 and 4 primarily focused on ongoing symptoms, whereas the in-person visit at week 12 included more comprehensive assessments using standardized instruments, including the Patient Health Questionnaire-9 and the Depression Anxiety Stress Scales-21. Accordingly, the present analysis captures acute hospitalization and short-term post-acute trajectories up to 12 weeks after discharge. Given the absence of longer-term follow-up, the dataset does not allow direct assessment of chronic long-COVID trajectories, delayed complications, or long-term functional recovery. This limitation should be considered when interpreting the persistence and clinical relevance of symptoms beyond the acute pandemic phase.

The Isfahan University of Medical Sciences (IUMS) ethics committee and the other national authorities approved the study (IR.MUI.RESEARCH.REC.1399.479). The experimental protocol was also conformed to the Declaration of Helsinki. Also, all the subjects gave written informed consent to the experimental procedure. The first relative family of patients with severe conditions gave the written informed consent. No minors participated in our study.

### 2.2 Exploratory data analysis

In this hospital-based prospective surveillance study, a total of 1998 patients with COVID-19 were admitted to the Khorshid Referral Hospital in Isfahan from February 2020 until September 2021. As part of data quality assurance, we excluded 61 patients who had neither any reported symptoms nor laboratory test results at admission. Following these exclusions, the final sample included 1937 patients. Table 2 presents a summary of demographic and admission-related variables of the study cohort. The mean age of patients was 58.66 years (SD = 16.51), and 58% were male. Average hospitalization lasted 7 days, with women admitted for 0.5 days less than men, and patients with underlying disease hospitalized 14 hours longer. The majority of patients were non-smokers (87.5%), married (94%), and employed (45.4%). Among those who underwent PCR testing, 76.1% tested positive for SARS-CoV-2. Admission to the intensive care unit (ICU) was required for 14.7% of patients, and 5% underwent endotracheal intubation. During the 12-week follow-up period, the all-cause mortality rate was 8.4%, with the vast majority of deaths (97.5%) occurring during hospitalization.

**Table 2:** Descriptive statistics of demographic and clinical characteristics of 1937 patients admitted to khorshid COVID-19 referral hospital, Isfahan (Feb 2020–Sep 2021).

Variable	Summary Statistics
Age (years)	Mean = 58.66, SD = 16.51
Hospitalization duration (days)	Mean = 7.16 days, SD = 5.48
	Frequency (Proportion)
Gender	Female: 814 (0.42) Male: 1123 (0.58)
Smoking status	No: 1749 (0.88) Yes: 248 (0.12)
Marital status	Single: 83 (0.04) Married: 1739 (0.94) Divorced/Widowed: 28 (0.02)
Occupation	Employed: 734 (0.45) Unemployed: 1203 (0.55)
PCR result	Positive: 1304 (0.76) Negative: 410 (0.24)
Quarantine before admission	Yes: 997 (0.89) No: 120 (0.11)
Underlying disease	Yes: 1261 (0.65) No: 676 (0.35)
ICU admission	Admitted: 284 (0.15) Not admitted: 1653 (0.85)
Intubation status	Yes: 97 (0.05) No: 1840 (0.95)
Mortality (12-week follow-up)	Alive: 1774 (0.92) Dead: 163 (0.08)
– in hospital	159 deaths
– after discharge	4 deaths

Tables 3 and 4 present the distribution of underlying disease and the prevalence of symptoms over time, respectively. Cardiovascular and metabolic disorders were the most frequently reported comorbidities, reported 46.3% and 39.6% of patients, respectively. During the 12-week follow-up period, the overall prevalence of symptoms declined; however, fever, fatigue, cough, and dyspnea remained the most persistent symptoms.

**Table 3:** Prevalence of underlying medical conditions among hospitalized COVID-19 patients in the KCC study.

Condition	Frequency	Proportion
Cardiovascular Disease	896	0.463
Metabolic Disease	767	0.396
Respiratory Disease	143	0.074
Renal Disease	128	0.066
Immunocompromised Disease	101	0.052
Autoimmune Disease	81	0.042

**Table 4:** Symptom prevalence at hospital admission and during 12-week follow-up among COVID-19 patients in the KCC study.

Symptom	Admission (W0)	Week 1 (W1)	Week 4 (W4)	Week 12 (W12)
Fever	0.707	0.0077	0.0041	0.0021
Chills	0.555	0.0062	0.0083	0.0072
Cough	0.709	0.1030	0.0790	0.0340
Shortness of Breath	0.625	0.0740	0.0860	0.0590
Runny Nose	0.097	0.0041	0.0067	0.0130
Sore Throat	0.162	0.0052	0.0057	0.0170
Nasal Congestion	0.036	0.0047	0.0031	0.0240
Sputum or Hemoptysis	0.047	0.0240	0.0220	0.0340
Nausea/Vomiting	0.389	0.0200	0.0150	0.0110
Diarrhea	0.240	0.0067	0.0036	0.0057
Decreased Appetite	0.425	0.0210	0.0250	0.0160
Fatigue/Weakness	0.562	0.0700	0.0830	0.0680
Weight Loss	0.165	0.0250	0.0520	0.0550
Chest Pain	0.249	0.0041	0.0230	0.0340
Headache/Dizziness	0.351	0.0077	0.0240	0.0310
Loss of Smell	0.118	0.0110	0.0160	0.0130

The laboratory test results exhibited an average missing data proportion of approximately 6.3%. In this dataset, the majority of missing values are associated with non-sensitive demographic variables. As such, the missingness mechanism is likely to be not at random (MNAR). Although the data may be MNAR, due to the low rate of missingness (<10%) and the dominance of MAR-consistent variables, we applied Multiple Imputation by Chained Equations (MICE), which has been shown to perform robustly under low levels of missingness across various MNAR scenarios, as demonstrated by Pereira et al. [26]. A summary of laboratory findings at the time of hospital admission is provided in Table 5. Reference ranges are based on the American Board of Internal Medicine (ABIM) Laboratory Test Reference Ranges (January 2025). Several notable abnormalities were observed, including elevated levels of C-reactive protein (CRP) and erythrocyte sedimentation rate (ESR), indicative of significant systemic inflammation. Lymphocyte counts were at the lower end of the normal range, consistent with the lymphopenia frequently reported in COVID-19 patients. Elevated aspartate aminotransferase (AST) levels suggested potential hepatic involvement. In addition, white blood cell (WBC) counts exhibited substantial variability (SD = 34.64), reflecting heterogeneous immune responses among patients.

**Table 5:** Laboratory results of COVID-19 patients at the time of hospital admission in the KCC study.

Test	Mean (SD)	Normal Range
Platelet count	196.38 (79.7)	150–450×10^9^/L
Neutrophil count	7.39 (1.16)	2–8.25×10^9^/L
Lymphocyte count	1.93 (1.02)	1.2-4.95×10^9^/L
Hemoglobin		
– Male	13.67 (2.01)	14-18 g/dL
– Female	12.5 (1.71)	12-16 g/dL
WBC	8.41 (34.64)	5–10×10^9^/L
CRP	57.84 (46.08)	≤0.8 mg/L
ESR		
– Male	43.33 (27.8)	0–15 mm/hr
– Female	47.48 (26.37)	0–20 mm/hr
AST	46.09 (41.77)	10-40 U/L
ALP	180.4 (87.2)	30-120 U/L
ALT	39.54 (100.88)	10–40 U/L
Creatinine		
– Male	1.42 (1.22)	0.7–1.3 mg/dL
– Female	1.17 (0.98)	0.5–1.1 mg/dL
BUN	19.54 (12.77)	8–20 mg/dL
Sodium (Na)	136.31 (4.12)	136–145 mEq/L
Magnesium (Mg)	1.96 (0.47)	1.6–2.6 mg/dL
Potassium (K)	4.02 (0.55)	3.5–5.0 mEq/L
Bicarbonate (HCO_3_)	25.17 (4.66)	23–28 mEq/L
PCO_2_	44.84 (10.76)	42-53 mm Hg
pH	7.38 (0.07)	7.32–7.41
Calcium (Ca)	8.54 (0.72)	8.6–10.2 mg/dL
Phosphate (P)	3.05 (0.84)	3-4.5 mg/dL

Table 6 summarizes medications used by patients prior to hospital admission, while Table 7 outlines pharmacological treatments administered during hospitalization. The analysis focused on the ten most frequently prescribed in-hospital treatments. Prior to admission, the most commonly used medications included diabetic agents (54%), insulin (44%), levothyroxine (44%), and antibiotics (0.53%). During hospitalization, the most frequently administered drugs were heparin (47%), methylprednisolone (44%), ceftriaxone (41%), and hydroxychloroquine (31%).

**Table 6:** Proportions of pre-hospital medication use among participants in the KCC COVID-19 cohort study.

Medication	Proportion	Medication	Proportion
Diabetic Agents	0.543	ICS	0.393
Antibiotics	0.527	Calcium	0.393
Insulin	0.441	Rosuvastatin	0.393
Levothyroxine	0.438	Rivaroxaban	0.390
PPIs	0.431	Digoxin	0.388
Acetaminophen	0.419	Diltiazem	0.388
Benzodiazepines	0.416	Methotrexate	0.387
Hydroxychloroquine	0.412	LAMA	0.386
SABA	0.411	Celecoxib	0.385
Antipsychotics	0.402	SAMA	0.385
Allopurinol	0.400	Calcitriol	0.384
Clopidogrel	0.398	Fibrates	0.384
H2 Blockers	0.395	Sulfasalazine	0.384
Montelukast	0.395	Memantine	0.384
Vitamin B	0.394	Cyclosporine	0.383
LABA	0.393	Lovastatin	0.383
Rivastigmine	0.383	Azathioprine	0.382

**Table 7:** The proportions of using different medications among COVID-19 patients enrolled in the KCC study during the hospitalization period.

Medication	Proportion	Medication	Proportion
Heparin	0.470	Warfarin	0.033
Methylprednisolone	0.444	Vitamin C	0.026
Ceftriaxone	0.408	Ciprofloxacin	0.018
Hydroxychloroquine	0.314	Co-Amoxiclav	0.014
Doxycycline	0.267	Clopidogrel	0.010
Enoxaparin	0.248	Metronidazole	0.009
Azithromycin	0.240	Calisthenic	0.008
Levofloxacin (tablet)	0.234	Cefepime	0.007
Tamiflu	0.148	Ribavirin	0.006
Linezolid	0.140	Rivaroxaban	0.006
Meropenem	0.137	Clindamycin	0.005
Vancomycin	0.108	Favipiravir	0.003
Interferon-β	0.097	Amikacin	0.002
Ceftazidime	0.083	Cotrimoxazole	0.001
Caltera	0.076	Cloxacillin	0.001
Aspirin	0.059	Cefixime	0.001
Tazocin	0.046	Naproxen	0.000

### 2.3 Methodology

This section outlines the methodological framework used to analyze the KCC longitudinal dataset. We begin by reviewing foundational concepts from graph theory and graphical models. Subsequently, we detail our extension of the mixture of DAGs framework to support multiscale data, incorporating both discrete and continuous variables in a unified setting.

#### 2.3.1 Graph theory.

A graph *G* = (*X*,*E*) consists of a set of vertices $X=\left(X_{1},...,X_{p}\right)$ and a set of edges *E*. The endpoints of edges can be of three types: tail, arrowhead, and circle. Each circle corresponds to an undetermined endpoint. The endpoint * is also used as a meta-symbol to represent a tail, an arrowhead, or a circle as an endpoint.

A directed graph is a graph that consists only of directed edges. In other words, the edge (*X*,*Y*) is distinct from the edge (*Y*,*X*). A graph is undirected if all its edges are of the form *X*–*Y*. The skeleton of a graph is the structure obtained by replacing all directed edges in the graph with undirected edges.

Two vertices in a graph are said to be adjacent if there is an edge (regardless of its type) between them. A path between two vertices is a sequence of consecutive edges (of any type) connecting the vertices, such that no vertex is visited more than once. A directed path between two vertices is a path consisting of directed edges that all follow the same direction (i.e., no two consecutive edges should both enter or both exit the same vertex). If there exists a directed path from $X_{i}$ to $X_{j}$, then $X_{i}$ is considered an ancestor of $X_{j}$, and $X_{j}$ is considered a descendant of $X_{i}$. Similarly, if there is a directed edge from $X_{i}$ to $X_{j}$, then $X_{i}$ is a parent of $X_{j}$, and $X_{j}$ is a child of $X_{i}$.

A cycle in a graph is a path that starts and ends at the same vertex without repeating any edges. A directed cycle occurs when there is a directed path from $X_{i}$ to $X_{j}$ and also a directed edge from $X_{j}$ to $X_{i}$. If there is a bidirected edge between $X_{i}$ and $X_{j}$, it forms an almost-directed cycle. A graph is acyclic if it contains no cycles. A directed graph is called a directed acyclic graph (DAG) if it contains no directed cycles.

#### 2.3.2 Graphical model for multiscale data.

Given a joint probability distribution and its corresponding DAG G, the independencies in the structure can be specified using the Markov assumption. This assumption states that each node is conditionally independent of all its non-descendants given its parents in the DAG G. A fundamental implication of this property is that the joint probability distribution p factorizes according to a DAG *G* if:


$$\begin{array}{c} p(X_{1},...,X_{p})=\prod_{i=1}^{p}p(X_{i}|Pa_{G}(X_{i})) \end{array}$$ (1)

where $Pa_{G}(X_{i})$ represents the set of parents of $X_{i}$ in the DAG G. While the factorization property provides a way to express independence through the structure of the DAG G, a more general graphical criterion for determining conditional independence is d-separation. It provides a systematic way to read off conditional independence relationships directly from the graph’s structure. Two vertices $X_{i}$ and $X_{k}$ are considered d-separated by a set of vertices **S** if specific structural conditions are met. D-separation occurs in two scenarios: If there is a path between $X_{i}$ and $X_{k}$ through a vertex $X_{j}$, and $X_{j}$ is in the conditioning set S, the path is blocked under these conditions:

-
$X_{i}\to X_{j}\to X_{k}$

-
$X_{i}\leftarrow X_{j}\leftarrow X_{k}$

-
$X_{i}\leftarrow X_{j}\to X_{k}$

In a collider path $X_{i}\to X_{j}\leftarrow X_{k}$, the path is blocked if $X_{j}$ and none of its descendants are in the conditioning set S. When all paths between $X_{i}$ and $X_{k}$ are blocked by S, the vertices are said to be d-separated, implying conditional independence given the variables in S.

The problem of causal discovery refers to the task of recovering the true graph $G^{*}$ from the given dataset [27]. An algorithm is constraint-based if it utilizes conditional independence (CI) testing to recover some aspects of $G^{*}$ as a consequence of the Markov property and faithfulness which is the converse of the Markov property.

In this paper, we extend the classical setting to multiscale data, where the random variable $X_{i}$ is either discrete or continuous. Let D and C denote the sets of discrete and continuos variables respectively. The joint probability distribution *p*(**X**) then factorizes as:


$$\begin{array}{c} p(\text{X})=\prod_{i\in \text{D}}p(X_{i}|{\text{Pa}}_{G}(X_{i}))\cdot \prod_{j\in \text{C}}p(X_{j}|{\text{Pa}}_{G}(X_{j})) \end{array}$$ (2)

For each node $X_{i}$, $Pa_{G}(X_{i})$ could be any type (discrete, continuous, or mixed). This is unlike a work like [28], which does not allow discrete variables to have continuous parents. The equation (2) can be re-written as:


$$\begin{array}{c} p(\text{X})=\prod_{i\in \text{D}}p(X_{i}|{\text{Pa}}_{G}^{\left(d\right)}(X_{i}),{\text{Pa}}_{G}^{\left(c\right)}(X_{i}))\cdot \prod_{j\in \text{C}}p(X_{j}|{\text{Pa}}_{G}^{\left(d\right)}(X_{j}),{\text{Pa}}_{G}^{\left(c\right)}(X_{j})) \end{array}$$ (3)

where $Pa_{G}^{\left(d\right)}\subseteq \text{D}⧵X_{i}$ and $Pa_{G}^{\left(c\right)}\subseteq \text{C}⧵X_{i}$ denote the discrete and continious parents of $X_{i}$, respectively.

### 2.4 The mixture of DAGs framework for multiscale data

The proposed causal discovery methodology for multiscale data is based on the mixture of DAGs framework. Unlike traditional methods that assume a single underlying causal structure for all samples, the mixture formulation allows for heterogeneity across units. In this framework, each observation is assumed to be drawn from one of several underlying DAGs, which may vary across different contexts such as time, location, or subpopulations. Formally, the joint distribution can be expressed as a mixture:


$$\begin{array}{c} p(\text{X})=\sum p(\text{T}=t)p(\text{X}|\text{T=t)}. \end{array}$$

For each component, the joint distribution factorizes according to a DAG, while the overall population distribution is represented as a mixture of such structures. This formulation provides a flexible way to model dynamic or context-specific causal processes, as commonly observed in longitudinal clinical data, where causal mechanisms may vary over time or across patient subgroups and disease stages.

The framework accounts for latent confounding, selection bias, and changing graphical structure — phenomena that are common in longitudinal medical datasets. For instance, selection bias can occur when follow-up data is only available for patients who survive or consent to continued monitoring, while latent variables may include unmeasured patient traits or hospital-level factors.

While CIM is designed for continuous data [16], clinical datasets typically include a mix of continuous and categorical variables (e.g., age, laboratory findings, disease symptoms status, severity of symptoms, underlying diseases, drug dosages). To accommodate this, we adapted the likelihood model used within CIM. This allows us to have the theoretical foundation of CIM while making it applicable to multiscale-type datasets.

To formalize this, consider a set of vertices Z=X∪T, where **T** is a set of mutually independent variables that index different causal structures. We partition **Z** into three non-overlapping sets of variables: the set of observed variables (**O**), latent variables (**L**) that are unmeasured but influence the observed system, and selection variables (**S**), which represent mechanisms that determine which units are included in the dataset or under what conditions measurements are recorded. The joint probability distribution *p*(**X**, **T**) is factorized as follows.


$$\begin{array}{c} p(\text{Z})=\prod_{i\in \text{D}}p(Z_{i}|{\text{Pa}}_{\text{T}}^{\left(d\right)}(Z_{i}),{\text{Pa}}_{\text{T}}^{\left(c\right)}(Z_{i}))\cdot \prod_{j\in \text{C}}p(Z_{j}|{\text{Pa}}_{\text{T}}^{\left(d\right)}(Z_{j}),{\text{Pa}}_{\text{T}}^{\left(c\right)}(Z_{j})) \end{array}$$ (4)

where $Pa_{\text{T}}(\text{T})=\emptyset$. This formulation allows the parent set $Pa_{\text{T}}(Z_{i})$ to vary based on the values of **T**, creating a mixture of different DAGs.

For each $Z_{i}\in \text{Z}$, let ${\text{U}}_{i}\subseteq \text{T}$ denote the largest set such that $U_{i}\cap Pa_{\text{T}}(Z_{i})=\emptyset$. This implies $\text{T}\cap Pa_{\text{T}}(Z_{i})=\text{T}⧵U_{i}\equiv V_{i}$. We can rewrite equation (4) to highlight which variables are stationary or non-stationary over subsets of **T**:


$$\begin{array}{cc} p(\text{Z}) & =\prod_{i\in D}p(Z_{i}|\left({\text{Pa}}_{\text{T}}^{\left(d\right)}(Z_{i}),{\text{Pa}}_{\text{T}}^{\left(c\right)}(Z_{i})⧵{\text{U}}_{i}\right)\cup {\text{V}}_{i})\\ & \times \prod_{j\in C}p(Z_{j}|\left({\text{Pa}}_{\text{T}}^{\left(d\right)}(Z_{j}),{\text{Pa}}_{\text{T}}^{\left(c\right)}(Z_{j})⧵{\text{U}}_{j}\right)\cup {\text{V}}_{j}) \end{array}$$ (5)

To incorporate the longitudinal structure of the data, we partition the observed variables into time-indexed subsets: $\text{O}=\left\{{\;}^{1}O,...{,}^{w}O\right\}$, where ${\;}^{k}O$ denotes the set of observed variables at *k* time step. It is assumed that causal influence flows forward in time — that is, variables at time step k may cause those at time $k^{{\;}^{\prime }}>k$, but not vice versa. We assume that we sample i.i.d. from the following marginal distribution $p(\cup_{k=1}^{w}O|\text{S})$:


$$\begin{array}{c} p(\cup_{k=1}^{w}O|\text{S})=\sum_{{\text{L}}_{d}}\int_{{\text{L}}_{c}}p(\cup_{k=1}^{w}O,{\text{L}}_{d},{\text{L}}_{c}|\text{S})\;d{\text{L}}_{c} \end{array}$$ (6)

where mixing occurs over T∩L if T∩L≠∅ and ${\text{L}}_{c}$ and ${\text{L}}_{d}$ are respectively latent variables for continuous and categorical variables.

To construct the graph structure, causal discovery algorithms rely on conditional independence (CI) tests between variables. However, classical CI tests are typically designed for either purely discrete or purely continuous data and are therefore not directly applicable to multiscale datasets. In the original CIM framework, Strobl [16] employs CI tests designed for continuous variables. For mixed-type data, we instead adopt the likelihood-based approach proposed by Andrews et al. [29]. Although originally developed within a score-based framework, the authors demonstrate that their scoring functions can be reformulated as conditional independence tests for use in constraint-based algorithms. This reformulation replaces Bayesian Information Criterion (BIC)-based model selection with likelihood ratio tests derived from the same probabilistic models. These tests are specifically designed for mixed-type variables and allow both discrete and continuous nodes to have arbitrary parent configurations, including continuous parents of discrete variables.

The corresponding model fitting procedure partitions the data according to configurations of discrete variables and fits conditional Gaussian models within each partition. The overall log-likelihood is computed as the sum of contributions across all partitions. For conditional independence testing, we use the Conditional Gaussian (CG) framework, implemented in the R package micd via the function mixCItest.

To formally test whether two variables $X_{i}$ and $X_{j}$ are conditionally independent given a set of conditioning variables $\text{Z}\subseteq \text{X}⧵\left\{X_{i},X_{j}\right\}$, we consider the hypotheses:


$$\begin{array}{c} \begin{array}{cc} H_{0} & :X_{i}⟂\;\;\;⟂X_{j}|\text{S}\\ H_{1} & :X_{i}\text{⧸}\;⟂\;\;\;⟂X_{j}|\text{S} \end{array} \end{array}$$

The test is constructed by comparing two models, a full model, where $X_{i}$ depends on $X_{j}$ and **Z** and a reduced model, where $X_{i}$ depends only on **Z**. The likelihood ratio test statistic is given by:


$$\begin{array}{c} G^{2}=-2\log(\frac{L_{reduced}}{L_{full}}) \end{array}$$

where $L_{full}$ and $L_{reduced}$ denote the likelihoods under the full and reduced (null) models, respectively. Under the null hypothesis, this test statistic *G*^2^ has a large-sample chi-squared distribution:


$$\begin{array}{c} G^{2}\sim \chi^{2}(df_{full}-df_{reduced}) \end{array}$$

where $df_{full}$ and $df_{reduced}$ are the degrees of freedom of the full and reduced models, respectively. A statistically significant result indicates that inclusion of $X_{i}$ significantly improves the model for $X_{i}$, leading to rejection of conditional independence.

### 2.5 Interpretability of a graph

The CIM algorithm returns a partially oriented mixed graph over the observed variables. A mixed graph may contain edges with arrowheads (>) and tails (–), while a partially oriented mixed graph extends this by allowing circles (°) at endpoints, indicating unresolved orientation. We begin with illustrative examples to elucidate key concepts and facilitate interpretation. For clarity and simplicity, all variables are assumed to occur within a single time step.

First, consider the true causal process, represented as a mixture of two DAGs in Fig 2a. Let the latent variable set be L={T}, and assume there is no selection variable, i.e., S=∅.

**Fig 2:**
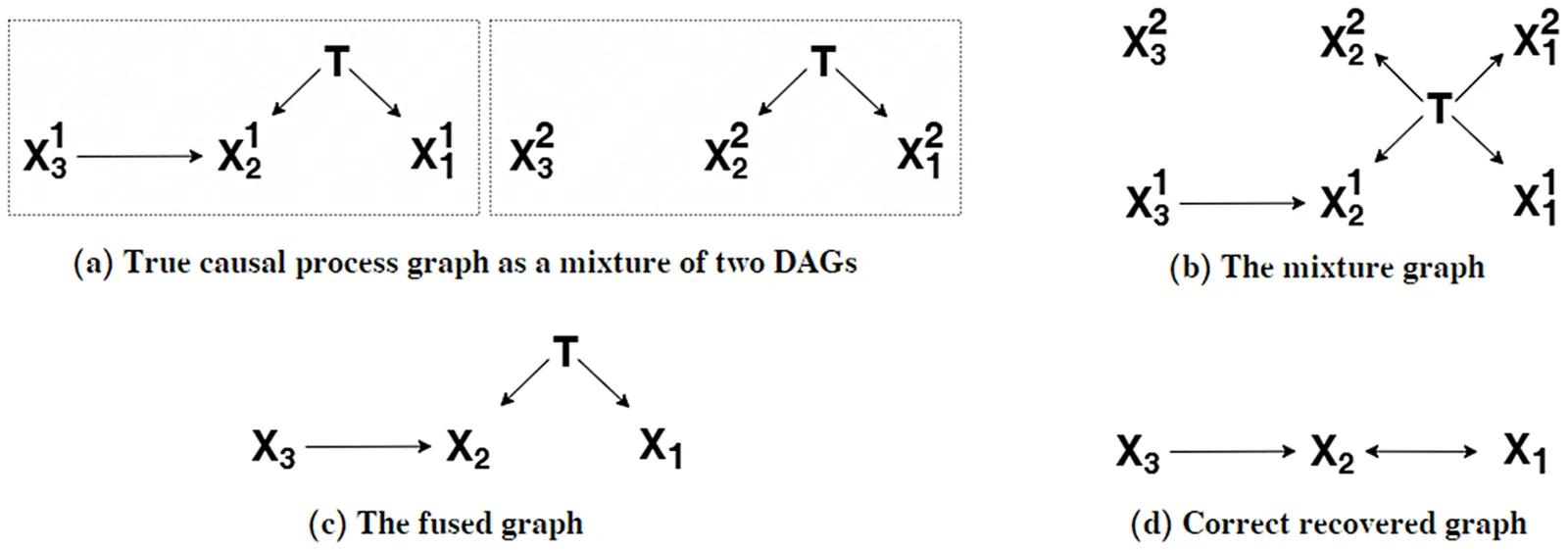
An example illustrating the influence of existence of a latent variable in the true causal process. The notation $X_{i}^{j}$ represents the variable $X_{i}$ associated with the j-th component graph of a causal process.

The mixture graph (Fig 2b) is constructed by placing the two component graphs adjacent to each other and then merging T into a single vertex. This structure is then fused into a single (potentially cyclic) graph (Fig 2c) by combining each set of corresponding variables across components, for example, $X^{{\;}^{\prime }}=\left\{X_{1}^{1},X_{1}^{2}\right\}$ is merged into a single variable *X*_1_. In the fused graph, *X*_2_ is not an ancestor of *X*_3_, there is no directed path from *X*_2_ to *X*_3_. This is reflected in the recovered graph (Fig 2d) by the presence of a directed edge from edge $X_{3}\to X_{2}$. Furthermore, while *X*_1_ and *X*_2_ are d-connected in the mixture graph, neither serves as an ancestor of the other. This relationship is indicative of a latent confounder influencing both variables. Accordingly, the bidirected edge in the recovered graph represents a statistical dependence without inferred causality in either direction.

To illustrate causal influence transmitted along a directed path, consider a modification of the previous example in which a latent variable *L*_1_ is added to the mixture of DAGs, yielding the latent variable set ($\text{L}=\left\{T,L_{1}\right\}$) (Fig 3a). The corresponding mixture graph and fused graph are shown in Fig 3b and 3c, respectively. In this setting, a directed path from *X*_2_ to *X*_1_ emerges in the fused graph via the latent variable *L*_1_. Since *X*_1_ is not an ancestor of *X*_2_, the recovered graph appropriately reflects this causal structure with a directed edge $X_{2}\to X_{1}$ (Fig 3d).

**Fig 3:**
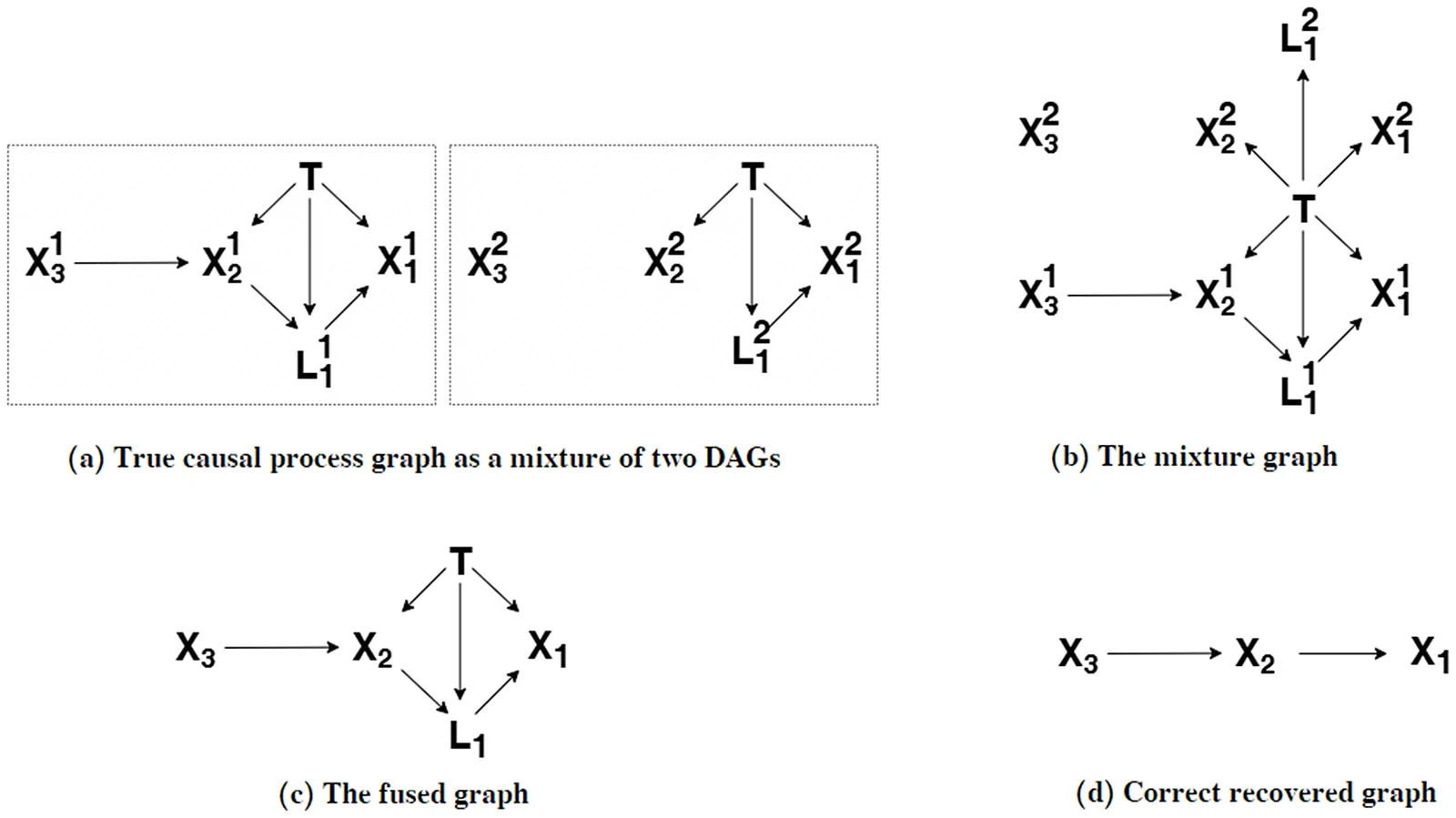
An example illustrating the interpretation of $X_{2}-*X_{1}$. The notation $X_{i}^{j}$ represents the variable $X_{i}$ associated with the j-th component graph of a causal process.

Next consider a hypothetical scenario that illustrates how selection bias can distort the interpretation of dependencies in observational data. Let *X*_1_ and *X*_2_ represent two rare symptoms within the general population, each arising independently from distinct disease processes, implying no underlying causal relationship. However, the way patients access care introduces a selection mechanism. If a patient exhibits either symptom *X*_1_ or *X*_2_, typically visit their medical doctor (MD), whereas those experiencing both *X*_1_ and *X*_2_ simultaneously — an unusual and concerning clinical presentation — are more likely to be referred to the emergency room (ER), where their case is recorded and included in the dataset. As a result, the dataset is implicitly conditioned on selection into the ER, which is more likely when both symptoms are present. This conditioning opens a collider path, where *S* represents selection into the ER. Although *X*_1_ and *X*_2_ are independent in the full population, they will appear statistically dependent in the selected dataset. In this case, the edge is shown as $X_{1}-X_{2}$, indicating that both *X*_1_ and *X*_2_ are inferred to have a directed path to the selection variable (Fig 4).

**Fig 4:**
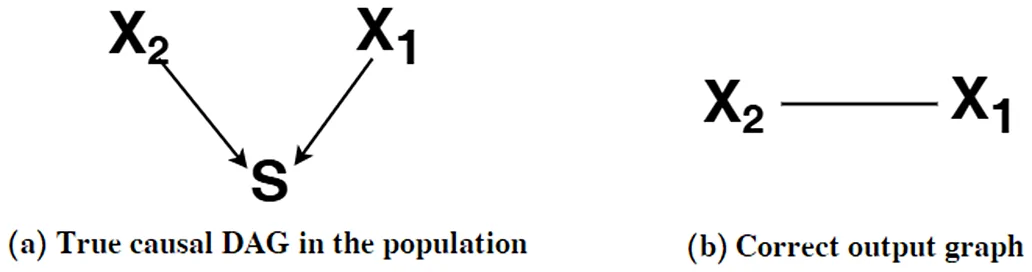
An example illustrating selection bias and its impact on the output.

These examples demonstrate how various edge types in the output graph reflect specific underlying causal or statistical relationships — arising from ancestral structure, latent confounding, or selection bias. To extend this understanding beyond specific cases, the CIM framework defines a set of interpretation rules that associate each edge type with a precise meaning. There are two key sets of rules:

1. Edge Presence or Absence (Adjacency):
  - The adjacency between two variables in the recovered graph means that these variables are not d-separated in the mixture graph by any subset of their adjacent variables or selection variables **S**.
  - Conversely, the absence of an edge implies that there exists at least one subset of adjacent or selection variables **S** that d-separates them.
2. Edge Endpoints (Orientation): For pairs of adjacent variables, CIM encodes ancestral relationships through the orientation of edge endpoints.
  - Having an arrowhead at $X_{j}$ in an edge $X_{i}*\to X_{j}$ means $X_{j}$ is not an ancestor of $X_{i}$ in the fused graph. In other words, when merging the component graphs of the true causal process, there is no directed path from $X_{j}$ to $X_{i}$.
  - Having a tail at $X_{j}$ in an edge $X_{i}*-X_{j}$ means $X_{j}$ is an ancestor of $X_{i}$ or of the selection variable set **S**, or both. This means, merging the component graphs of the true causal process, creates a directed path from $X_{j}$ to $X_{i}\cup \text{S}$.

In contrast to algorithms such as FCI, the interpretation of arrowheads in CIM does not incorporate selection variables. This is due to the practical limitation that, in many real-world scenarios, prior knowledge or wave information is insufficient to determine whether a given variable is an ancestor of the selection variables **S** within the fused graph.

## 3 Result

In this section, we present the main causal structures identified by the causal discovery algorithm. To ensure robustness, we performed 50 bootstrap iterations using 90% subsampling without replacement. Edges that appeared in at least 80% of the iterations were retained in the final graph. The results are organized into two parts: (1) cross-sectional analysis based on data collected at the time of hospital admission, and (2) longitudinal analysis capturing symptom progression, treatment effects, and outcome-related causal relationships. To guide interpretation of the edge types, Table 8 summarizes the common edge types identified by the algorithm. Each row characterizes a distinct relationship pattern with example instances and interpretive guidance. For selected findings, we perform follow-up statistical analyses to further investigate and validate the discovered associations. Fig 5 illustrates the proposed framework, which integrates causal discovery, graph-based interpretation, and effect quantification. Beginning with a multiscale longitudinal dataset, stable causal structures are identified using a causal discovery algorithm combined with bootstrap-based stability selection. These structures are then interpreted to reveal clinically meaningful relationships and generate hypotheses, which are subsequently evaluated through targeted causal inference techniques.

**Table 8:** Representative edge types observed in the causal discovery recovered graph.

Category	Description	Example(s)
Symptom Progression	Same symptom across time; persistent directionality.	CoughW0 → CoughW1
		CoughW1 *→ CoughW4
*Suggests a candidate causal persistence or evolution of symptoms. While reverse causality is ruled out, latent confounding may still explain the relationship.*		
Symptom Cascade	Early symptom leads to distinct symptom.	SoreThroatW0 *→ LossOfSmellW12
*Supports a plausible forward causal pathway. While reverse causality is ruled out, latent confounding may still explain the association.*		
Symptoms Within-Timestep	Two symptoms observed in the same time step with no clear causal direction.	ChillsW0 ∘−∘ FeverW0
*Indicates dependence. The algorithm finds no conditioning set to separate them; causal direction remains unresolved.*		
Symptom – Underlying Disease	Symptom appears to follow or be explained by an underlying disease.	Respiratory Diseases *→ DyspneaW0
*Direction supports clinically expected causality. The algorithm rules out reverse direction, though latent confounding cannot be ruled out entirely.*		
Admission Symptom – Treatment	Symptom observed at admission is linked to subsequent treatment decision.	WeightLoss0 *→ Hydroxychloroquine
*Likely reflects confounding from underlying severity; follow-up symptoms may not indicate a direct effect of cumulative treatment exposure.*		
Follow-up Symptom – Treatment	Symptom status during follow-up is linked to administered treatments.	Hydroxychloroquine ↔ CoughW1
*Likely reflects confounding from treatment assignment. May indicate side effects to be explored with further adjustment.*		
Treatment – Outcome	Clinical outcomes potentially affected by treatments.	Death ↔ Ceftriaxone,
		Heparin ↔ ICU Admission
*Likely reflects confounding by indication; causal effect may be estimated with further adjustment.*		
Underlying Disease – Treatment	Chronic conditions and assigned treatments are statistically dependent.	Cardiovascular Diseases ↔ Aspirin
*Reflects clinical protocols; often structurally confounded.*		
Treatment – Treatment	Treatments commonly co-administered or appear together in practice.	Vancomycin ↔ Ceftazidime
*Cumulative dosages of treatments appear statistically associated, likely reflecting co-prescription protocols or shared clinical indications.*		
Outcome – Outcome	Clinical outcomes (Death, ICU Admission, and Intubation Status) are linked together.	Death Status ↔ Intubation Status
*Represents strong statistical association between outcomes, likely due to confounding.*		

*Note:* W0 denotes symptoms at admission (baseline), while W1, W4, and W12 corresponds to follow-up at 1, 4, and 12 weeks post-discharge, respectively.

**Fig 5:**
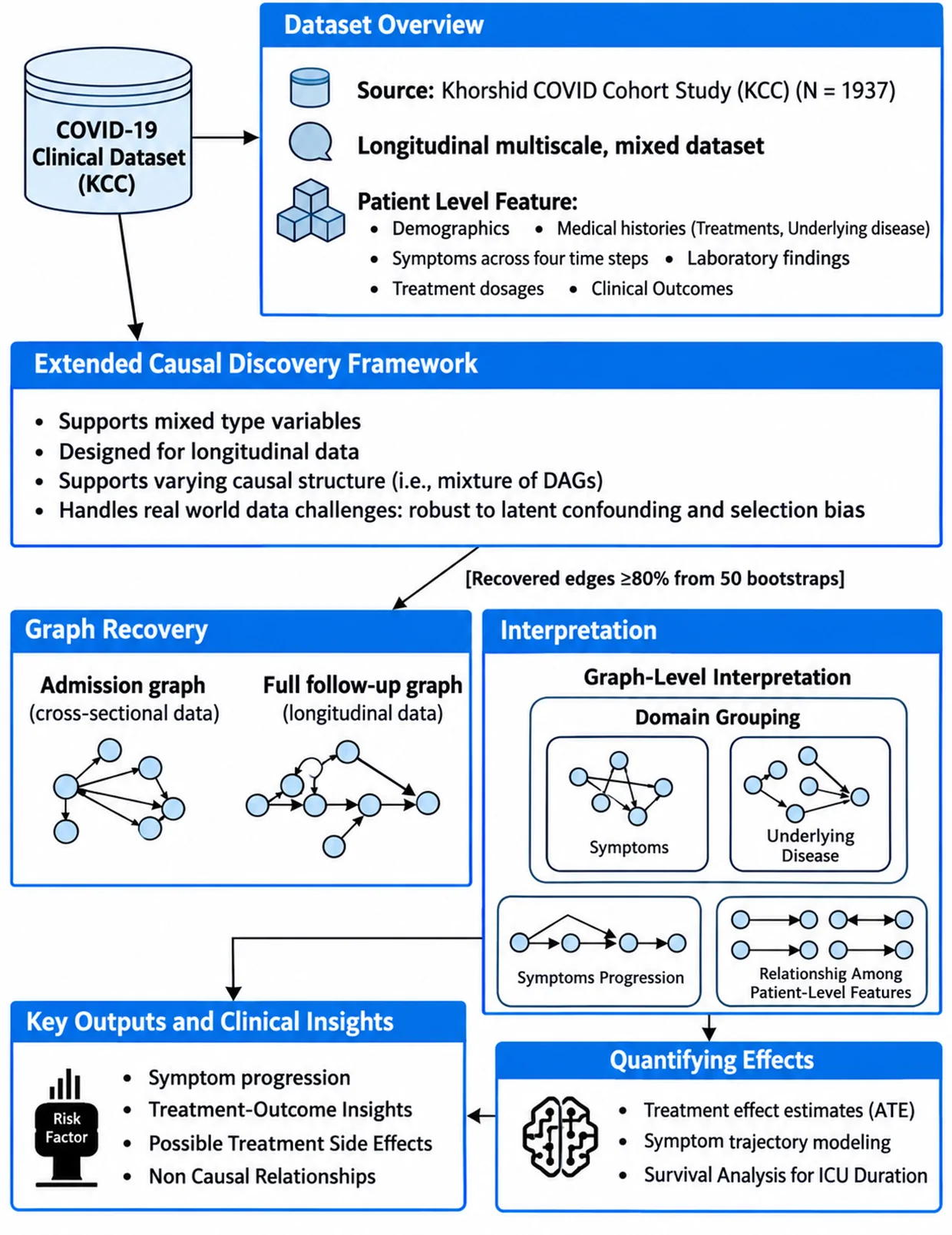
Proposed framework of this study applied to the KCC longitudinal dataset.

The goal is to understand how pre-existing conditions and symptoms at admission cluster and interact, how symptoms evolve over time, and how treatments administered during hospitalization may influence–or appear to influence–subsequent clinical outcomes (death, intubation status, or leading to ICU), laboratory markers, and also prolongation of COVID-19 symptoms. Special attention is given to distinguishing genuine causal effects from statistical associations driven by latent variables or selection mechanisms. This multi-layered strategy enables a nuanced application of causal discovery to uncover plausible mechanisms that shape the progression and management of COVID-19 in real-world clinical settings.

### 3.1 Admission time

In the first part of our analysis, we analyze patients’ medical histories, symptoms at admission, laboratory findings, and underlying diseases. The recovered graph by the algorithm is presented in Fig 6. Since CIM is specifically designed for longitudinal studies, and this section utilizes cross-sectional data collected at admission, we do not infer edge orientations. Instead, our interpretation focuses solely on variable adjacencies–whether two variables are connected in the undirected skeleton graph. Any interpretation of causal relationships must be approached with caution, particularly due to the direction of causation and the potential presence of latent confounders and selection bias. Nevertheless, the skeleton graph still offers valuable insights.

**Fig 6:**
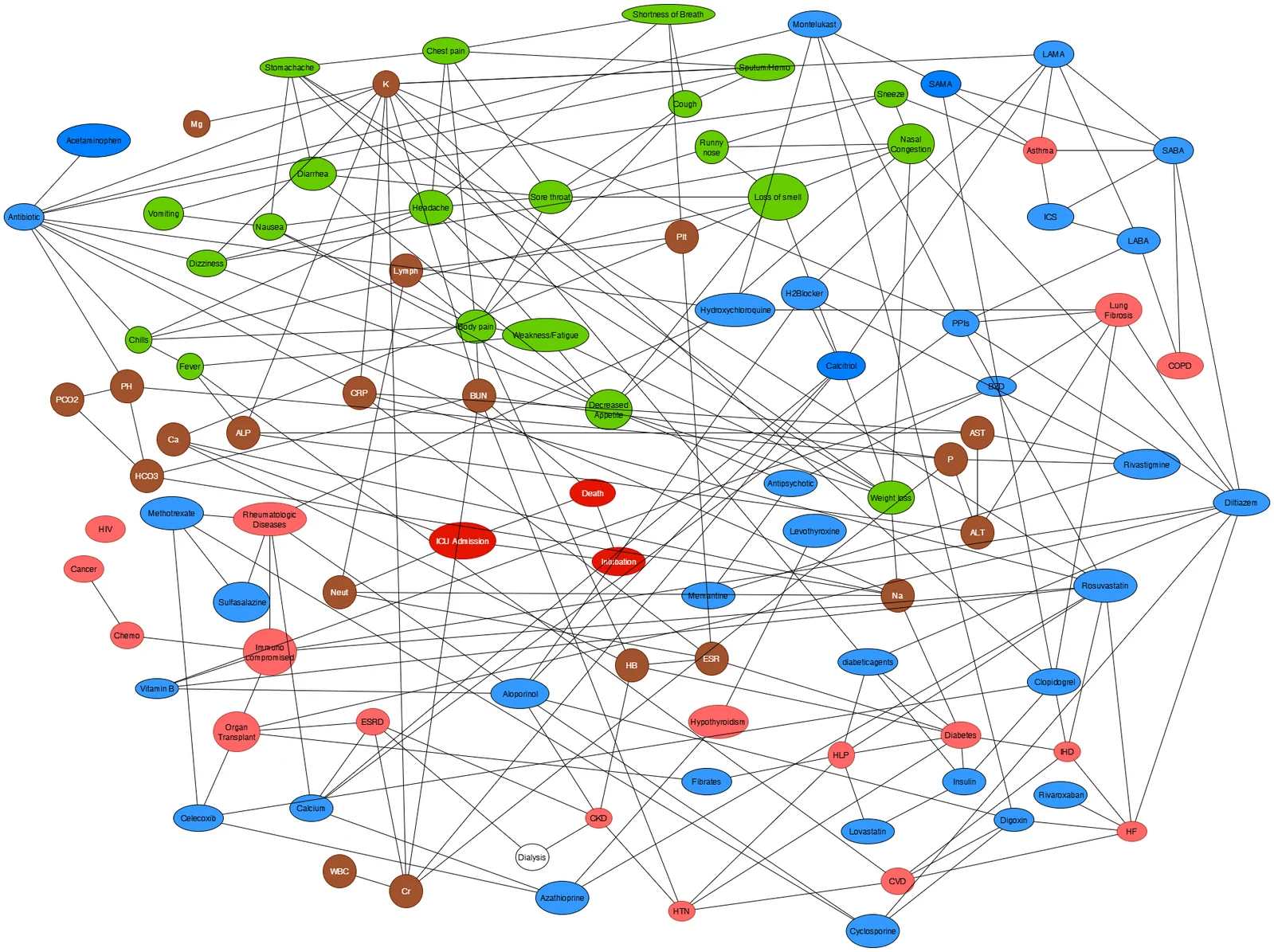
Graph of COVID-19 using a cross-sectional dataset with green nodes (symptoms at admission), pink nodes (medical histories), blue nodes (prior medications), brown nodes (laboratory findings at admission) and red nodes (clinical outcomes such as death, intubation status or leading to ICU).

#### 3.1.1 Domain-specific groupings at admission.

The graph reveals visually distinct groupings of symptoms (Fig 7) and underlying diseases (Fig 8) among COVID-19 patients. In the symptom-level graph, a close relationship is seen among both upper respiratory symptoms–such as nasal congestion, sneezing, sore throat, runny nose, and loss of smell–and lower respiratory symptoms, including cough, shortness of breath, and sputum/hemoptysis. This reflects the shared involvement of the respiratory tract in COVID-19. A second grouping consists of gastrointestinal symptoms, including nausea, vomiting, diarrhea, and stomachache, highlighting the involvement of the gastrointestinal system. Additionally, systemic symptoms formed another distinctive grouping characterized by constitutional symptoms like fever, chills, and weakness/fatigue, and body pain which were closely linked with metabolic indicators such as weight loss and decreased appetite. In the disease-level graph, several established groupings of patients’ underlying diseases are evident. These include cardiovascular conditions (CVD, HTN, IHD, HF), metabolic disorders (Diabetes, HLP, Hypothyroidism), renal diseases (CKD, Dialysis, ESRD), and immunosuppression-related conditions (Immunocompromised status, Organ Transplant, Cancer, Chemotherapy). While these graphs highlight strong within-group relationships, care should be taken when interpreting the absence of edges. For instance, although Asthma, COPD, and Lung Fibrosis appear unconnected in the disease-only graph, their associations may emerge more clearly in the full graph (Fig 6) when symptoms and treatments are considered. Similarly, not all observed links imply direct relationships; some may arise from shared underlying causes.

**Fig 7:**
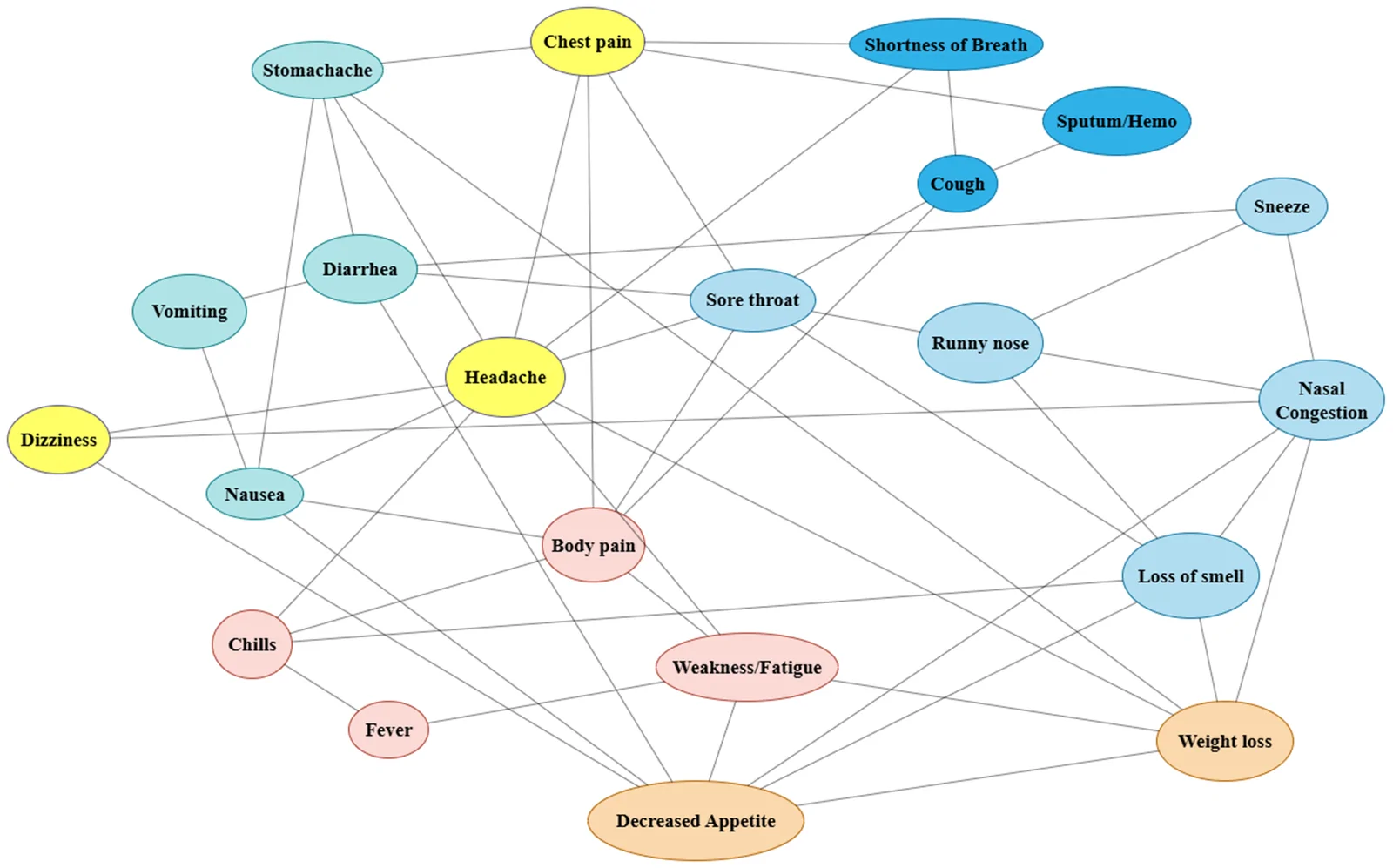
Domain-based grouping of COVID-19 symptoms at admisson among KCC study patients.

**Fig 8:**
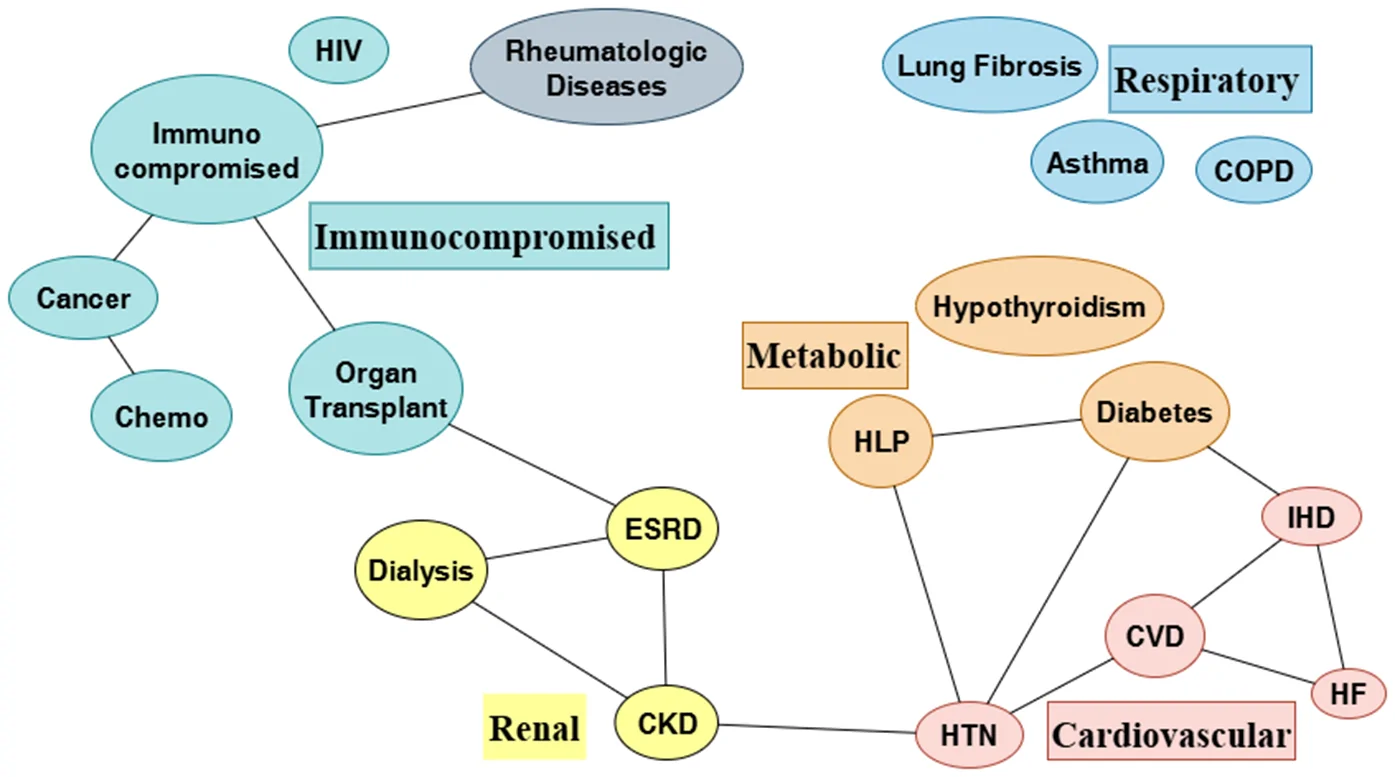
Domain-based grouping of underlying diseases among COVID-19 patients in the KCC study.

#### 3.1.2 Cross-domain relationships and clinical outcomes.

Beyond the within-domain patterns, the graph also highlights several cross-domain relationships. These edges reflect how clinical presentation, underlying diseases, laboratory findings, and medical histories are intertwined in real-world patient data. For example some medications can be seen adjacent to related diseases, reflecting their clinical use in managing those conditions, and some are also connected to each other, suggesting they may be commonly prescribed together. In the recovered graph, only a few variables remain adjacent to outcomes. Notably, blood urea nitrogen (BUN) is linked to death, while neutrophil count and calcium are connected to ICU admission. Additionally, death, ICU admission, and intubation status form a tightly connected triad.

Taken together, these relationships provide a structural overview of how symptoms, underlying diseases, treatments, laboratory findings, and outcomes are connected at the time of hospital admission. While the graph is based on cross-sectional data and does not permit any direction, it highlights meaningful associations.

### 3.2 Longitudinal analysis

In this section, we leverage the longitudinal structure of the KCC dataset. First, we explore causal relationships between symptoms during 12-week follow-up among hospitalized patients during the COVID-19 outbreak, along with their medical histories (categorized into six groups based on the grouping of the first part of the study), laboratory findings at admission, cumulative dosages of treatments administered during hospitalization, and medical outcomes. The sequence of repeated symptoms for each patient, recorded at four time points (admission and weeks 1, 4, and 12 post-discharge), enables us to study symptom progression, treatment efficacy, and potential biases. To guide interpretation, Table 8 summarizes key edge types. The Causal inference from graph-derived hypotheses subsection presents quantifying effects guided by graph-derived hypotheses.

#### 3.2.1 Graph-based insights.

This section reports the findings of the causal discovery analysis regarding longitudinal symptom progression, treatment effects, and outcome-related structures.

**Symptom progression over time** We initiated our analysis by examining the temporal progression of symptoms across time. The causal graph recovered by the algorithm (Fig 9) reveals that several symptoms persist across multiple time steps while others dissipate more quickly. Additionally, not all symptoms strictly follow a sequential transition (i.e., edges of the form $symp_{t}\to symp_{t+1}$). Symptoms such as cough, shortness of breath, weakness/fatigue, weight loss, and loss of smell are observed to continue through later follow-ups. These findings align with those from our previous association-based study [18].

**Relationships across clinical domains** The recovered graph reveals a range of cross-domain connections linking symptoms, treatments, laboratory findings, underlying diseases, and clinical outcomes. For instance, treatments appear adjacent to both symptoms and outcomes–highlighting possible therapeutic effects, side effects, or confounding by disease severity. Blood urea nitrogen (BUN) is linked to mortality, while treatments such as Heparin or Interferon-β are linked to ICU-related outcome. Additionally, several treatments like Vancomycin and Ceftazidime appear frequently co-administered, as reflected by their statistical associations. Some of these findings align with prior clinical understanding. For instance, although Interferon-β has been explored as a treatment for severe COVID-19, its observed link to ICU admission likely reflects confounding by disease severity–patients in more critical condition are both more likely to receive Interferon-β and require ICU care. The graph correctly captures this confounding structure by identifying a shared common cause [30]. Importantly, the algorithm does not interpret the relationships among the clinical outcomes themselves–such as ICU admission, intubation status, and mortality–as direct causal links. Instead, it suggests that the relationships among these outcomes are much more complex. A summary of common edge types–including treatment–outcome, treatment–symptom, and symptom progression–is provided in Table 8, which offers interpretations and representative examples drawn from the graph.

**Fig 9:**
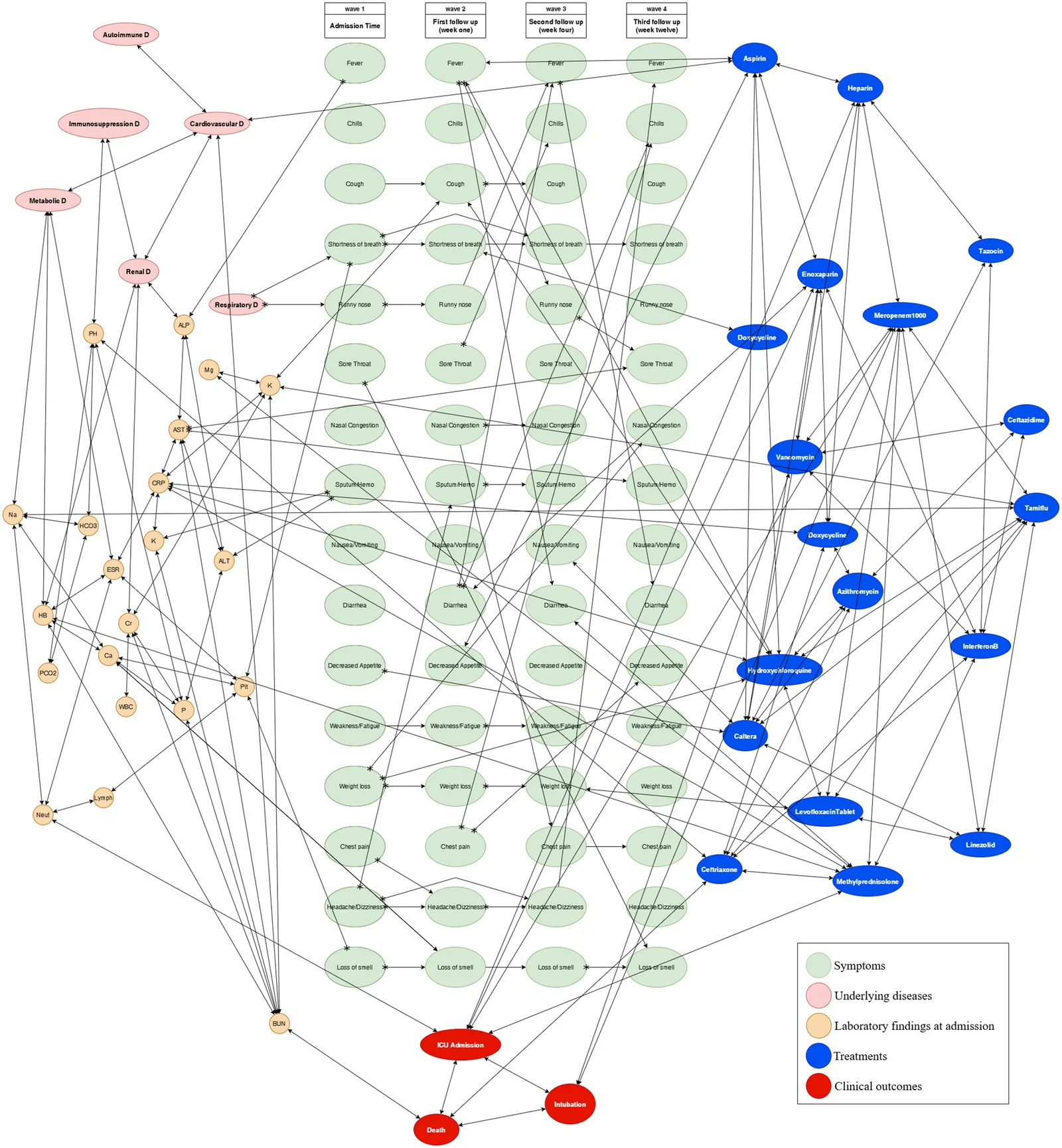
Causal graph of COVID-19 using a longitudinal dataset with symptoms over four time steps (green nodes), underlying diseases (pink nodes), laboratory findings at admission (brown nodes), treatments given during hospital stay (blue nodes), and clinical outcomes (red nodes).

#### 3.2.2 Causal inference from graph-derived hypotheses.

Based on the interrelations identified in the graph, we now analyze specific hypotheses informed by these connections. The causal discovery algorithm identified multiple potential causal relationships between cumulative dosages of treatments, symptoms, and clinical outcomes. To investigate these hypothesized relationships, we conducted setting-specific follow-up analyses using appropriate models aligned with our study objectives and the nature of the outcome. Specifically, to estimate average treatment effects (ATE) for treatment dosages, we used a weighting approach based on generalized propensity scores (GPS), implemented via the CausalGPS package in R [31]. GPS extends conventional propensity score methods to accommodate continuous treatment variables, addressing limitations of binary-focused approaches [32]. Covariate balance was assessed using standardized mean differences (SMD), with a threshold of 0.1 applied to confirm adequate balance post-weighting.

The following subsections present the findings from these targeted analyses. For selected graph-derived hypotheses, we compared unadjusted regression, multivariable-adjusted regression, and generalized propensity score–adjusted models. These analyses were not intended to demonstrate the superiority of the proposed method over existing approaches; rather, they were used to assess whether graph-derived relationships remained stable after adjustment for clinically relevant confounders and whether observed associations were attenuated after accounting for treatment-selection mechanisms. Accordingly, the results are interpreted as robustness checks and hypothesis-generating evidence, rather than as definitive validation of the proposed algorithm.

**Evaluating symptom progression** We examined the extent to which symptoms present at admission could predict their persistence and evolution at 1, 4, and 12 weeks post-discharge. Relationships identified through a causal discovery algorithm were further examined using logistic regression models, adjusting for clinical severity and other relevant covariates. Table 9 summarizes the predictive effects of baseline and intermediate symptoms on their presence at subsequent follow-ups. Notably, symptoms such as cough, shortness of breath, and loss of smell demonstrated strong temporal continuity, suggesting well-defined progression patterns over time. For example, logistic regression modeling with shortness of breath at week 1 as the outcome and baseline shortness of breath as the primary predictor revealed that patients presenting with the symptom at admission had 3.03 times higher odds of reporting it at week 1 compared to those without shortness of breath at baseline.

**Table 9:** Adjusted logistic regression results for five symptoms of “cough”, “shortness of breath (SOB)”, “fatigue”, “weight loss” and “loss of smell” presence at 1, 4, and 12 Weeks post-discharge, controlling for clinical severity and relevant covariates.

Outcome Symptom	Predictor(s)	OR (95% CI)	p-value
CoughW1	CoughW0	4.09 [2.55, 6.97]	<0.001
CoughW4	CoughW1, CoughW0	6.82 [4.18, 11.11], 2.72 [1.42, 5.23]	<0.001
SOBW1	SOBW0	3.03 [1.78, 5.15]	<0.001
SOBW4	SOBW1, SOBW0	4.31 [2.82, 6.48], 3.61 [2.29, 5.64]	<0.001
SOBW12	SOBW4	16.37 [11.21, 23.62]	<0.001
FatigueW1	FatigueW0	2.55 [1.73, 3.78]	<0.001
FatigueW4	FatigueW1	6.87 [4.34, 10.7]	<0.001
WeightLossW1	WeightLossW0	1.95 [1.01, 3.70]	0.0441
WeightLossW4	WeightLossW1	0.18 [0, 3.52]	0.003
LossofSmellW1	LossofSmellW0	5.02 [1.88, 12.39]	0.001
LossofSmellW4	LossofSmellW1	5.35 [1.15, 24.5]	0.03
LossofSmellW12	LossofSmellW4	9.87 [1.78, 55.94]	0.01

*Note:* W0 denotes symptoms at admission (baseline), while W1, W4, and W12 correspond to follow-up at 1, 4, and 12 weeks post-discharge, respectively.

**Treatment effect analysis** Treatment exposure was represented using cumulative in-hospital dose as a single non–time-varying variable, given the availability of detailed medication timing and total administered dose in the KCC dataset. However, cumulative dose is not a purely exogenous treatment measure. It may be influenced by length of hospital stay, survival time, clinical deterioration, early treatment discontinuation, physician decision-making, and evolving hospital protocols. Consequently, cumulative dose may partly reflect disease severity or the opportunity to receive treatment rather than the biological effect of the medication itself. To avoid overinterpretation, all treatment-effect analyses were considered exploratory. Whenever feasible, models were adjusted for clinical severity and compared with generalized propensity score–based approaches. In the presence of detailed time‑varying exposure data, time-varying exposure models, marginal structural models, or joint models of treatment initiation, treatment duration, and outcome would be more appropriate for definitive causal estimation and should be considered in future work.

***Effect of Ceftriaxone on mortality*** For the graph-derived relationship between ceftriaxone and mortality, we fitted logistic regression models with death status as the binary outcome and cumulative ceftriaxone dosage as the main exposure. The unadjusted model showed a negative association between cumulative ceftriaxone dose and mortality, and this association remained after adjustment for severity and after generalized propensity score adjustment (Table 10). However, this finding should not be interpreted as evidence that ceftriaxone directly reduces COVID-19 mortality. Ceftriaxone is an antibacterial agent, and its use may reflect suspected or documented bacterial co-infection, hospital treatment protocols, physician selection, clinical severity patterns, or other unmeasured factors. In addition, cumulative dose may be affected by survival time and length of hospitalization. Therefore, the observed association is best interpreted as a graph-guided, hypothesis-generating signal requiring cautious clinical interpretation and external validation.

**Table 10:** Comparison of ceftriaxone effect estimates with and without GPS adjustment.

Analysis	Odds Ratio	95% CI	P-Value
(Logistic Regression), unadjusted	0.914	[0.871, 0.953]	<0.001
(Logistic Regression), adjusted	0.897	[0.854, 0.937]	<0.001
GPS	0.835	[0.801, 0.971]	0.03

We also explored interactions between ceftriaxone and five co-administered treatments identified through the causal discovery graph, including Methylprednisolone, Tamiflu, Azithromycin, Interferon-β, and Doxycycline. Among these exploratory interaction terms, only the ceftriaxone–interferon-β interaction reached nominal statistical significance. The direction of the coefficient suggested that the negative association between Ceftriaxone and mortality may be attenuated among patients receiving higher doses of Interferon-β. However, the effect estimate was small, the confidence interval was close to the null, and no external replication was available. Therefore, this interaction should not be interpreted as evidence of a clinically established adverse interaction. It should instead be considered an exploratory signal that may reflect residual confounding, co-treatment patterns, severity-based prescribing, or chance finding due to multiple testing.

***Effect of treatments on ICU duration*** In the causal discovery graph, five pharmacologic agents, Heparin, Methylprednisolone, Enoxaparin, Meropenem, and Interferon-β, were directly connected to ICU admission. While the graph dentified an association between ICU admission as a binary outcome, evaluating the effect of treatments on ICU length of stay among admitted patients offers a more comprehensive assessment of treatment impact. Therefore, for patients admitted to the ICU, we modeled ICU duration as a time-to-event outcome using Cox proportional hazards regression. All models were adjusted for clinical severity to account for its potential confounding effect.

Overall, the estimated effects of most treatments remained consistent across modeling approaches. Both Heparin and Interferon-β were significantly associated with prolonged ICU stays in all analyses. Methylprednisolone exhibited a modest yet stable effect. Notably, the association between Meropenem and ICU duration reached statistical significance only in the GPS-weighted model. Enoxaparin showed no significant relationship with ICU duration in any of the model. Interaction effects were generally non-significant, with the exception of a significant interaction between Interferon-β and Linezolid (p = 0.01, HR = 0.9989, 95% CI: [0.9981, 0.9998]).

***Effect of treatments on symptom persistence at follow-up*** To explore possible treatment effects on symptom trajectories, we evaluated selected drug–symptom relationships (Table 11). All treatment–symptom pairs appeared statistically significant in unadjusted or adjusted models but lost significance under the GPS framework. This suggests that once confounding is properly addressed, there is no evidence of a meaningful treatment effect.

**Table 11:** Cumulative treatment dosage effects on follow-up symptoms (OR, 95% CI, p-value).

Outcome Symptom	Treatment	Model	OR [95% CI]	p-value
DiarrheaW4	Methylprednisolone	Unadj	1.000 [1.000–1.001]	0.01
		Adj	1.000 [1.000–1.001]	0.009
		GPS	1.05 [0.75, 1.47]	0.786
FeverW1	Aspirin	Unadj	1.15 [1.008–1.27]	0.006
		Adj	1.000 [1.025–1.288]	< 0.001
		GPS	1.15 [0.57, 2.30]	0.7
NauseaVomitingW4	Caltera	Unadj	1.03 [1.002, 1.05]	0.01
		Adj	1.03 [1.002, 1.06]	0.01
		GPS	1.08 [0.89, 1.31]	0.458

*Note:* W0 denotes symptoms at admission (baseline), while W1, W4, and W12 correspond to follow-up at 1 and 4weeks post-discharge, respectively.

## 4 Simulation study

### 4.1 Experimental setup

To evaluate the performance of the proposed method in mixed-type longitudinal settings, synthetic datasets were generated using a mixture of DAGs framework. The simulation framework was designed to capture heterogeneous and non-stationary causal processes, in which the underlying causal structure may vary across samples and over time. The data generation process was repeated over 50 independent iterations. In each iteration, a new mixture of DAGs was instantiated, and a longitudinal dataset comprising *n* = 2000 observations was generated according to the corresponding data-generating process. The overall simulation workflow is outlined in Algorithm 1.

**Algorithm 1 Simulation Framework for Data Generation and Evaluation**

**Phase I: Construction of the Mixture of DAGs**

1:  Initialize an empty DAG over *p* variables partitioned into *w* waves

2:  Sample edge indicators independently from Bernoulli(d/(p−1))

3:  Add temporal edges between consecutive waves

4:  Assign variable types (binary or continuous)

5:  Draw number of mixture components $n_{T}$ and mixing probabilities $P(T_{i})$

6:  Partition the graph edges among components via block randomization

7:  Select latent variables **L** and selection variables **S**

**Output:** Mixture of DAGs with component probabilities, latent variables, and selection mechanism

**Phase II: Data Generation**

8:  **for** each observation i=1,...,n **do**

9:   Activate each component independently according to its mixing probability; let *A* denote the resulting active graph

10:   **for** each node *j* in topological order of *A* **do**

11:    **if** *j* is continuous **then**

12:     $x_{j}\sim 𝒩\;\left(\sum_{k\in {Pa}_{A}(j)}\beta_{kj}\;x_{k},\;1\right)$

13:    **else**

14:     $x_{j}\sim Bernoulli\;\left(\sigma \;\left(\sum_{k\in {Pa}_{A}(j)}\beta_{kj}\;x_{k}\right)\right)$

15:    **end if**

16:   **end for**

17:  **end for**

18:  Filter observations according to the selection variables

19:  Remove latent variables from the generated dataset

**Output:** Observed mixed-type longitudinal dataset of size *n*

**Phase III: Evaluation**

20:  Apply CIM, PC, FCI, RFCI, and CCI to the observed dataset

21:  Compare estimated ancestral relations to the ground-truth graph

22:  Compute sensitivity, fallout, balanced accuracy, overall score, and runtime

**Output:** Performance metrics per algorithm

The proposed method was compared against several constraint-based causal discovery algorithms shown in Fig 1, including PC, FCI, RFCI, and CCI. All algorithms were implemented in R using identical conditional independence tests and a significance level of α=0.01. To ensure a fair comparison across methods in a longitudinal setting, all competing algorithms were provided with time-step (wave) information during the skeleton discovery phase in order to orient arrowheads between the time steps. This prevents artificial performance degradation arising from the absence of explicit temporal ordering. In the absence of such information, all competing methods exhibited substantially weaker performance due to difficulties in resolving cross-time causal directions.

### 4.2 Data-generating process

Synthetic data generation consisted of two stages: construction of the mixture of DAGs and sampling mixed-type longitudinal observations from the resulting causal process.

Let $\text{X}=\{X_{1},...,X_{p}\}$ denote the set of variables, where *p* = 64 variables are organized into four waves of 16 variables each. The underlying causal process was represented as a mixture of DAGs. We represent the collection of DAGs over **X** for the underlying causal process as $𝒢=\left\{{𝔾}^{1},{𝔾}^{2},...,{𝔾}^{q}\right\}$.

To construct each mixture of DAGs, an adjacency structure was first constructed. The adjacency structure was represented as an undirected skeleton $G_{S}=(𝐗,E)$, where $E_{ij}$ is an adjacency indicator such that $E_{ij}=1$ if and only if there exists an edge $X_{i}-X_{j}$, and $E_{ij}=0$ otherwise. Edge indicators were sampled as


$$\begin{array}{c} E_{ij}\sim \text{Bernoulli}\left(\;\frac{d}{p-1}\right),\;i<j \end{array}$$

with $E_{ji}=E_{ij}$, yielding a random undirected graph with expected neighborhood size *d*. To preserve temporal ordering, directed edges were additionally inserted between corresponding variables across consecutive waves, creating persistence links that capture repeated measurements over time. Specifically, if variable $X_{j}$ appeared in consecutive waves, directed edges were added from wave *t* to wave *t* + 1. Random edge coefficients were sampled from


$$\begin{array}{c} \beta_{ij}\sim \text{Uniform}(1,2) \end{array}$$

and assigned random signs, resulting in a weighted linear DAG.

Let $𝐓=\{T_{1},...,T_{r}\}$ denote the set of mutually independent binary component indicators. Each component indicator $T_{i}$ was assigned a mixing probability $\pi_{i}=P(T_{i}=1)$. The graph edges were partitioned among the *r* graph components using block randomization, where the number of components *r* varied across simulation runs.

Variable types were assigned at the level of the original 16-variable template and propagated consistently across all four waves so that repeated measurements retained the same variable type over time.

For each observation, component indicators were sampled independently according to their mixing probabilities, and the active graph, denoted by *A*, was constructed as the union of edge blocks associated with the activated components (i.e., those with $T_{i}=1$).


$$\begin{array}{c} P(\text{T}=t)=\prod_{i=1}^{r}P(T_{i}=t_{i}), \end{array}$$

The active graph was constructed as the union of edges sets associated with components for which $T_{i}=1$. Variables were subsequently generated according to the topological ordering of the active graph *A*. Continuous variables were sampled from a linear Gaussian conditional distribution:


$$\begin{array}{c} X_{j}|{Pa}_{A}(j)\sim 𝒩\;\left(\sum_{k\in {Pa}_{A}(j)}\beta_{kj}X_{k},\;1\right), \end{array}$$

whereas binary variables were generated from a Bernoulli distribution:


$$\begin{array}{c} X_{j}|{Pa}_{A}(j)\sim Bernoulli\;\left(\sigma \;\left(\sum_{k\in {Pa}_{A}(j)}\beta_{kj}X_{k}\right)\right), \end{array}$$

where $\sigma (z)=(1+e^{-z}{)}^{-1}$ denotes the logistic function, and ${Pa}_{A}(j)$ represents the parent set of $X_{j}$ in the active graph *A*.

A total of 2000 observations were generated in each iteration. To mitigate potential ordering effects, variable ordering was randomized prior to algorithm execution.

### 4.3 Simulation scenarios

The proposed framework was evaluated under two simulation scenarios designed to differ in terms of latent confounding and selection bias.

**Scenario 1 (Absence of latent variables or selection bias).** In the first scenario, neither latent variables nor selection bias were incorporated into the data-generating process:


$$\begin{array}{c} \text{L}=\emptyset ,\;S=\emptyset \end{array}$$

where **L** denotes the set of latent variables and **S** represents the set of selection variables. Consequently, all variables were fully observed, and causal sufficiency was assumed.

**Scenario 2 (Latent confounding and selection bias).** In the second scenario, latent confounding and selection bias were explicitly introduced into the data-generating process. Candidate latent variables were selected from nodes having at least two children in the graph. Let L⊂X denote the set of latent variables. Selection variables were subsequently chosen from nodes outside the latent-variable set that had at least two parents:


$$\begin{array}{c} \text{S}\subset \text{X}⧵\text{L}. \end{array}$$

Variables assigned to **L** were removed from the dataset before analysis to create partially observed data. Selection bias was then introduced by filtering observations according to the selected variables prior to constructing the final observed sample. This scenario was designed to emulate violations of causal sufficiency and selection mechanisms that frequently arise in real-world longitudinal settings.

### 4.4 Metrics and results

All simulation studies were performed on the cloud computing platform of Ferdowsi University of Mashhad using a virtual machine configured with 32 vCPUs, 64 GB RAM, and 100 GB storage running Windows 10. The analyses were conducted using R version 4.6.0.

Figs 10 and 11 display the simulation results for Scenario 1 and Scenario 2, respectively, and summarize the comparative performance of the methods considered in this study.

**Fig 10:**
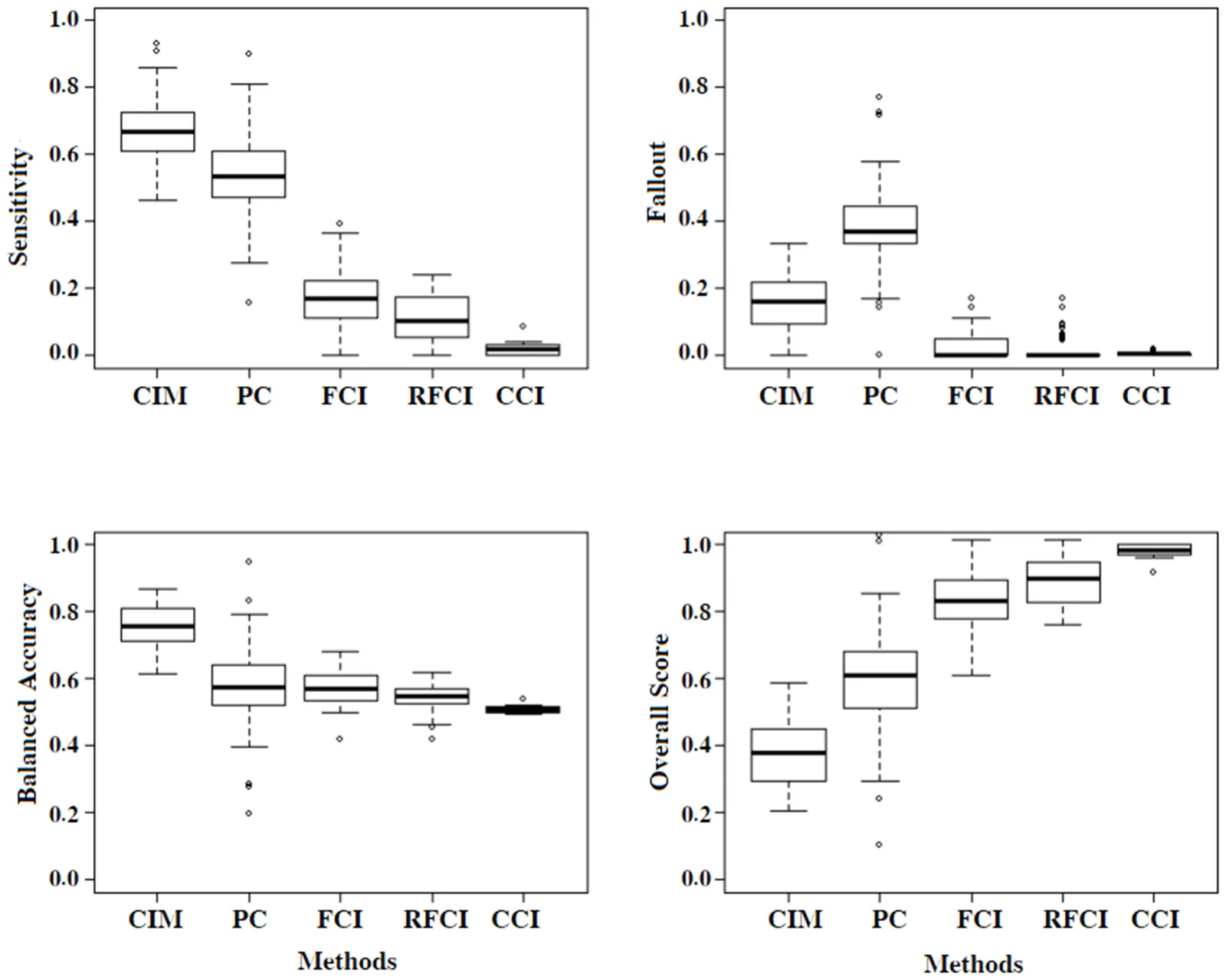
Performance comparison of CIM, PC, FCI, RFCI, and CCI under Scenario 1 across sensitivity, fallout, balanced accuracy, and overall score.

**Fig 11:**
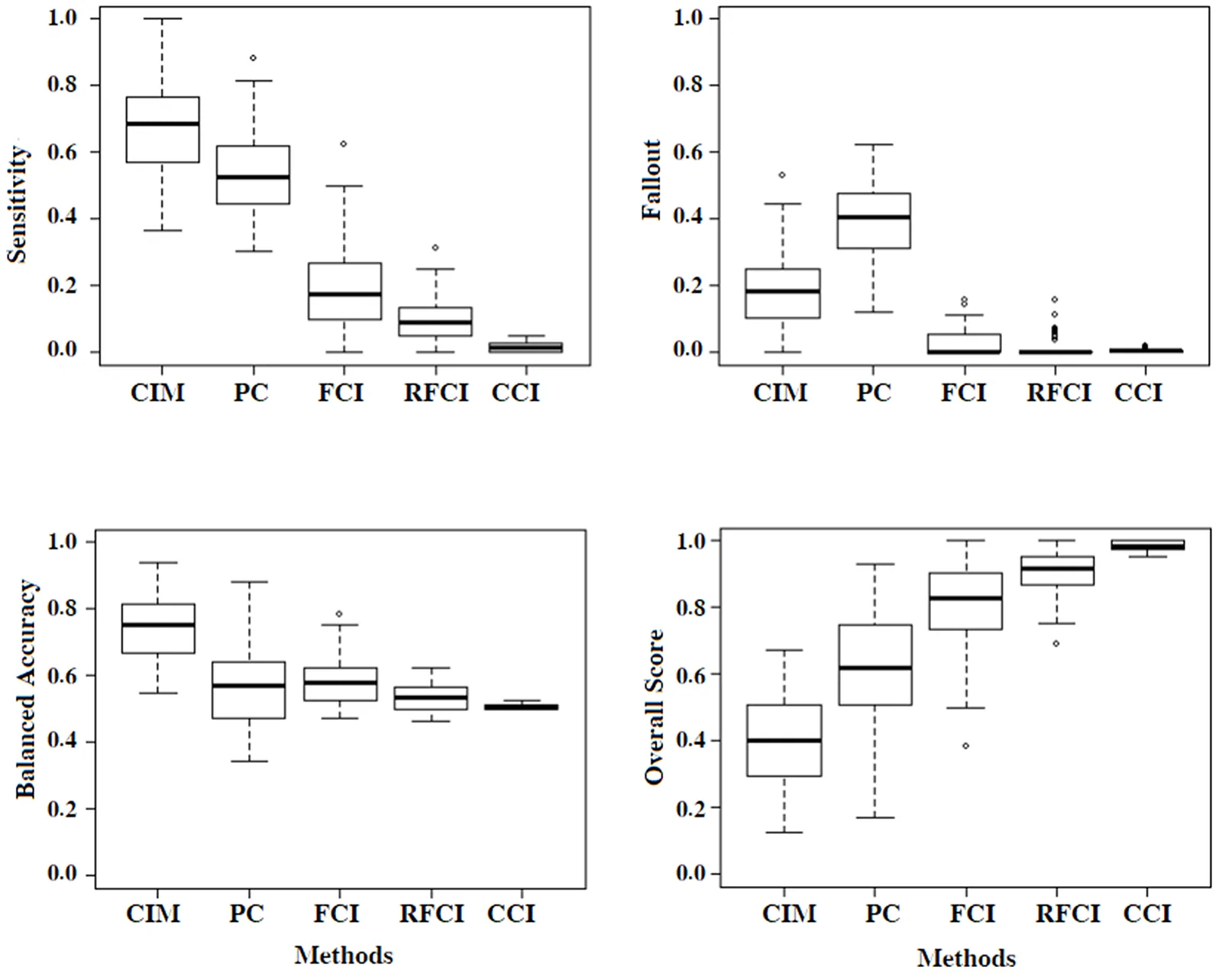
Performance comparison of CIM, PC, FCI, RFCI, and CCI under Scenario 2 across sensitivity, fallout, balanced accuracy, and overall score.

CIM was compared against PC, FCI, RFCI, and CCI. All methods were provided with identical wave membership information to ensure consistent between-wave orientation. Results were aggregated over 50 independent simulation runs.

Performance was evaluated using four metrics. Let *TP*, *FP*, *TN*, and *FN* denote the number of correctly and incorrectly inferred ancestral relations between pairs of nodes in the true and estimated graphs. Specifically, true positives correspond to correctly recovered ancestral relations in the ground-truth graph, while false negatives denote missed ancestral relations. False positives correspond to incorrectly inferred ancestral relations, and true negatives represent correctly identified non-ancestral relations. Sensitivity is defined as


$$\begin{array}{c} \text{Sensitivity}=\frac{TP}{TP+FN}, \end{array}$$

which measures the proportion of true ancestral relations correctly recovered. Also, Fallout is defined as


$$\begin{array}{c} \text{Fallout}=\frac{FP}{FP+TN}, \end{array}$$

which quantifies the proportion of falsely inferred ancestral relations among all non-ancestral node pairs. Balanced accuracy is defined as


$$\begin{array}{c} \text{BA}=\frac{1}{2}\left(\text{Sensitivity}+\text{Specificity}\right), \end{array}$$

where $\text{Specificity}=\frac{TN}{TN+FP}$. The overall score is computed as the Euclidean distance from the ideal point in ROC space:


$$\begin{array}{c} \text{Score}=\sqrt{(1-\text{Sensitivity}{)}^{2}+{\text{Fallout}}^{2}}, \end{array}$$

where lower values indicate better performance.

Figs 10 and 11 present a comparative evaluation of CIM, PC, FCI, RFCI, and CCI under Scenarios 1 and 2, respectively, in terms of sensitivity, fallout, balanced accuracy, and overall score. The boxplots summarize the distribution of each performance metric across 50 independent Monte Carlo simulation runs, thereby illustrating both the central tendency and variability of the algorithms’ performance.

Across both scenarios, CIM achieved the highest sensitivity, consistently recovering a larger number of true ancestral relations than the competing methods. Although the sensitivity distributions of CIM and PC showed some overlap, the median sensitivity of CIM remained higher throughout the simulation runs. In contrast, FCI, RFCI, and CCI recovered relatively few true ancestral relations, resulting in substantially lower sensitivity.

Importantly, the superior recovery performance of CIM was not accompanied by an increase in false discoveries. In both scenarios, CIM maintained relatively low fallout and exhibited a considerably lower false-positive rate than PC. While PC attained moderate sensitivity, this was achieved at the expense of substantially higher fallout. The comparatively low fallout observed for FCI, RFCI, and CCI appears to stem from their conservative edge-orientation behavior rather than from effective recovery of ancestral relations.

The boxplots further demonstrate that CIM not only delivered the strongest overall performance but also maintained a relatively stable level of performance across repeated simulations. Although some variability was observed, particularly under Scenario 2 where latent confounding and selection bias were present, the balanced-accuracy distribution of CIM remained clearly separated from those of the competing methods. Consequently, CIM achieved the highest balanced accuracy and the lowest overall score in both scenarios, placing it closest to the optimal region of the ROC space. Finally, the runtime analysis indicates that CIM requires approximately the same computational effort as PC and RFCI, whereas FCI and CCI are substantially more computationally expensive (Fig 12).

**Fig 12:**
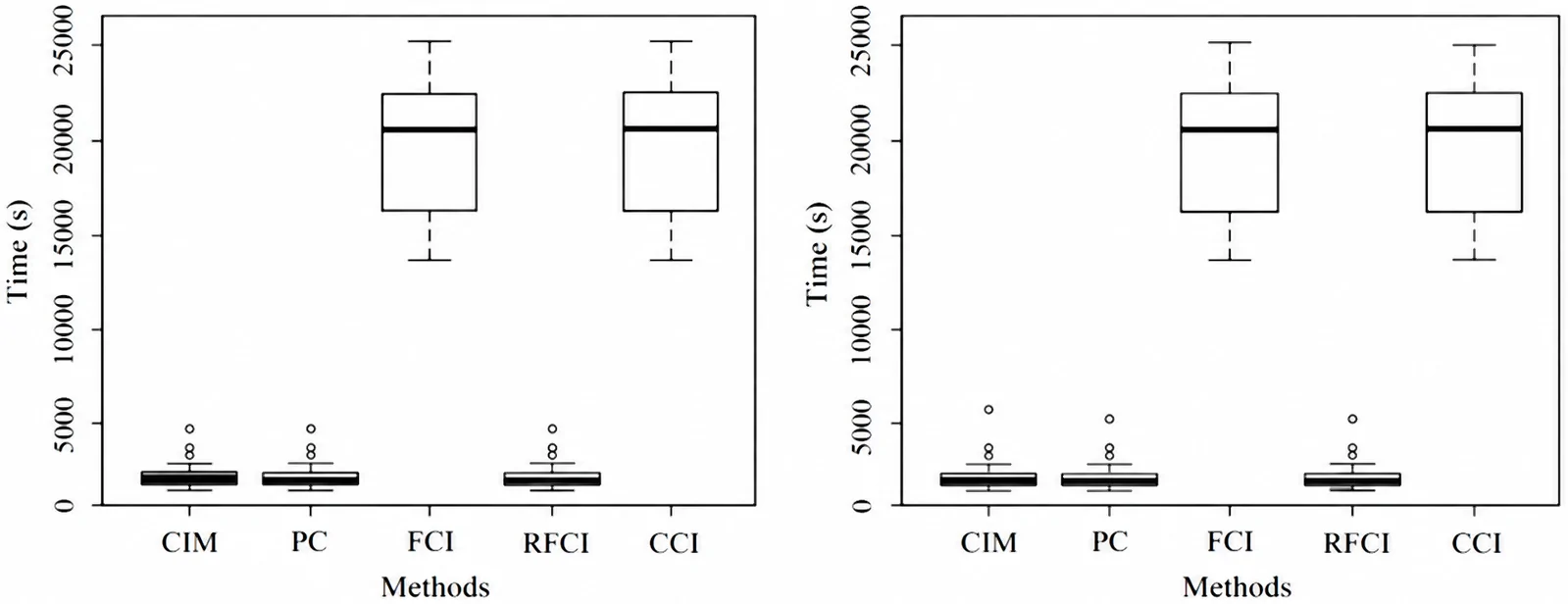
Boxplot comparison of execution times for CIM, PC, FCI, RFCI, and CCI across 50 simulation runs under Scenarios 1 and 2.

## 5 Discussion

In this study, we extended the CIM framework to accommodate mixed-type longitudinal clinical data and applied it to a large cohort of hospitalized COVID-19 patients with follow-up extending to 12 weeks after discharge. The analysis recovered clinically coherent admission symptom clusters, short-term persistence of selected post-acute symptoms, and several graph-derived relationships linking treatments, laboratory measures, and outcomes. The primary contribution of this work lies in demonstrating how an extended mixture of DAG framework can organize complex longitudinal clinical data and highlight candidate structural relationships that are more likely robust to confounding and selection mechanisms. The findings should nevertheless be interpreted cautiously. The cohort reflected early-pandemic clinical practice, follow-up was limited to 12 weeks, and no independent external cohort was available for replication. Therefore, the results are best viewed as feasibility evidence and hypothesis-generating clinical signals rather than definitive causal conclusions.

Among the most notable findings was the persistence of symptoms such as cough, shortness of breath, fatigue, and loss of smell, during the post acute period, highlighting the heterogeneous nature of symptom trajectories across patients and showing partial consistency with previous studies [18].These patterns should be interpreted as short-term post-acute trajectories rather than evidence of long-term post-COVID outcomes or chronic functional impairment, given the 12-week follow-up limitation. The observed symptom persistence is clinically plausible; however, these findings are presented primarily as evidence that the proposed framework can recover clinically expected longitudinal structures rather than as novel clinical discoveries.

Our analysis of treatment effects revealed notable insights with potential clinical relevance. Ceftriaxone was associated with lower mortality, and this relationship persisted after adjustment using generalized propensity scores (GPS). However, a significant interaction between Ceftriaxone and Interferon-β suggested that this association may be attenuated among patients receiving higher doses of Interferon-β. Similarly, treatments such as Heparin, Interferon-β, Methylprednisolone, and Meropenem showed associations with longer ICU stays. Our analysis also provided important insight into treatment-related side effects. Although several follow-up symptoms initially appeared associated with specific treatments, rigorous confounding adjustment using GPS suggested that many of these relationships were more likely attributable to confounding than to direct treatment effects. However, these findings require particularly cautious interpretation. For instance, ceftriaxone may have been prescribed to patients with suspected bacterial co-infection, to patients treated under specific hospital protocols, or to patients whose clinical course allowed longer treatment exposure. Conversely, patients who died early may have had less opportunity to receive higher cumulative doses. These mechanisms may generate an apparently protective association even in the absence of a direct causal effect. Although adjustment and generalized propensity score analyses reduced some concerns, residual confounding and time-related biases remain plausible explanations for part of the observed associations. Accordingly, these findings should be interpreted as graph-derived signals and hypothesis-generating evidence rather than definitive evidence of treatment effects. We therefore explicitly do not interpret the observed inverse association between ceftriaxone and mortality as evidence of a causal protective effect. Instead, this relationship requires further evaluation using time-varying treatment models, marginal structural models, and independent datasets. The treatment-related findings should also be interpreted in the historical context of the study period. Also, the cohort was recruited during the early phases of the pandemic, before widespread vaccination and before the emergence of later variants. Treatment protocols during this period differed substantially from current COVID-19 management, and some medications used in this cohort, including hydroxychloroquine and interferon-β, are no longer routinely used in many contemporary clinical settings. Therefore, treatment–outcome relationships identified in this study should not be directly generalized to current clinical practice. Their primary relevance is methodological, illustrating how graph-based approaches may help identify treatment-selection patterns, co-prescription structures, and potential sources of confounding in complex observational datasets.

Routine observational clinical data are subject to multiple sources of uncertainty, including incomplete recording, measurement error, evolving documentation practices, unmeasured clinical judgment, and protocol changes over time. In the KCC dataset, some variables may have been measured only when clinically indicated, and missingness may not be completely random. Although multiple imputation and bootstrap-based stability selection may reduce analytic instability, they cannot eliminate bias arising from unmeasured confounding, informative missingness, or heterogeneous documentation practices. Consequently, the apparent precision of graph structures and estimated effects should not be interpreted as evidence of correctly identified causal mechanisms. Instead, the proposed framework should be viewed primarily as a tool for organizing complex clinical data and generating clinically interpretable hypotheses rather than as a substitute for rigorous epidemiological design, external validation, or randomized evidence.

An important methodological contribution of this study is its use of causal discovery algorithms to guide variable selection and hypothesis formulation for subsequent causal inference analyses. This framework directly addresses a noted shortcoming in COVID-19 literature, where a recent review found that only 26.32% of COVID-19 studies using propensity score methods provided a clear rationale for variable selection [33] The proposed graph-based framework provides a reasonable justification for covariates selection. Overall, the framework applied here shows promise not only for COVID-19 but also for other diseases with complex temporal dynamics and heterogeneous data types.

This study represents a real-world feasibility application rather than a full validation of the proposed mixed-type CIM extension. Although the framework is able to recover clinically coherent and stable graph structures, these outputs do not guarantee identification of the true underlying causal graph. In general, causal discovery methods require validation under simulation settings with known ground truth, systematic comparison with alternative algorithms, and controlled evaluation under violations such as latent confounding, selection bias, and missingness mechanisms. Given that this study is based on a single observational cohort, the recovered structures should therefore be interpreted as data-driven, model-dependent hypotheses rather than validated causal relations. Future work should address these limitations through simulation-based benchmarking, replication in independent cohorts, and formal comparison with established epidemiological approaches.

This study has several important limitations. First, the follow-up period was limited to 12 weeks after discharge, restricting inference on long-term post-COVID outcomes, chronic long-COVID trajectories, delayed complications. Second, the analysis was based on a single early-pandemic hospital cohort, and may not reflect current treatment practices following vaccination and viral evolution. Third, although simulation-based benchmarking with known ground truth was conducted and demonstrated robust methodological performance under controlled conditions, the inferred graph structures and treatment-effect findings have not been externally validated in independent clinical cohorts. Consequently, the generalizability of these findings to other populations, healthcare systems, and stages of the pandemic remains uncertain. Fourth, the observational nature of the data limits causal interpretation. Although the framework is designed to address heterogeneity, latent confounding, and selection mechanisms more flexibly than many traditional approaches, it cannot fully eliminate residual bias arising from observational data and evolving clinical practice. Fifth, cumulative treatment dose may reflect length of stay, survival time, disease severity, treatment discontinuation, and physician decision-making, introducing time-related bias. Therefore, treatment-effect estimates should be interpreted as exploratory signals. Future studies should incorporate time-varying exposure models, marginal structural models, target trial emulation, and external validation. Finally, the findings should be interpreted as hypothesis-generating rather than confirmatory evidence of causal effects.

Future research should integrate clinical expertise more systematically into the proposed framework to improve interpretability and robustness of causal discovery. In addition, since the current study was conducted using a single dataset, further work is needed to investigate the generalizability of the estimated causal effects across different populations and clinical settings. The framework of causal transportability [34] provides a principled framework for evaluating when causal conclusions can be transferred across settings and may enhance external validity. Future work should also incorporate time-varying exposure information to better capture dynamic treatment effects. In such settings, methodological approaches such as time-varying exposure models, marginal structural models, and joint models of treatment and outcome processes may provide more reliable causal estimates.

In conclusion, this study highlights the value of using a longitudinal causal discovery framework to understand the complex temporal and treatment-related dynamics of COVID-19. Our findings may inform targeted clinical management strategies and contribute methodologically to the broader field of causal inference in healthcare research. This work provides a methodological foundation for broader applications of causal discovery in multiscale clinical data and supports the development of explainable AI methods in longitudinal medical datasets.

## 6 Conclusion

This study extended the mixture of DAGs framework to accommodate mixed-type longitudinal clinical data and applied it to a large cohort of hospitalized COVID-19 patients. The proposed approach identified clinically coherent admission clusters, short-term symptom-persistence patterns, and graph-derived relationships among treatments, laboratory measures, and outcomes. These findings support the feasibility and practical utility of using an extended mixture of DAG framework to organize complex clinical trajectories and generate interpretable, graph-informed hypotheses. However, these findings should be interpreted cautiously. The study was limited to 12 weeks of follow-up, relied on a single early-pandemic cohort, lacked independent external validation, and used observational treatment data that remain susceptible to confounding and time-related biases. Consequently, the results should not be interpreted as definitive evidence of treatment effects or long-term post-COVID mechanisms. Future work should emphasize replication in independent cohorts, evaluation across diverse clinical settings, comparison with conventional epidemiological approaches, and the application of time-varying causal models for more rigorous treatment-effect estimation.

Collectively, these findings suggest that extended mixture of DAG frameworks may provide a useful tool for organizing complex longitudinal clinical data and prioritizing hypotheses for future clinical investigation.

## Supporting information

S1 Appendix R Implementation of the Simulation Framework and Causal Discovery Workflow. This appendix provides the R implementation used throughout the simulation study and methodological evaluation presented in this work, supporting reproducibility of the proposed framework and analyses.
(PDF)
