# Supplementary material for: Causal discovery and inference in COVID-19: A multiscale longitudinal analysis using mixtures of DAGs
Source: PLoS One. 2026 Oct 30;21(10):e0355553. doi: 10.1371/journal.pone.0355553 (PMC13722330; doi:10.1371/journal.pone.0355553)
Supplement: Supplementary file 1 [file pone.0355553.s001.pdf]

S1 Appendix: R Implementation of the Simulation Framework and Causal Discovery Workflow

This appendix provides the R implementation used throughout the simulation study and methodological evaluation presented in this work, supporting reproducibility of the proposed framework and analyses

```r
# ------------------------------------------------------------
# Step 0: Load required libraries
# ------------------------------------------------------------
library(devtools)
library(graph)
library(processx)
library(RBGL)
library(pcalg)
library(CIM)
library(CCI)
library(micd)

# ------------------------------------------------------------
# Step 1: Generate a mixture of DAGs
# ------------------------------------------------------------

generate_mix_DAGs <- function(p, en, waves) {

  # Sample upper-triangular adjacency structure
  N       <- p * p - p
  samples <- rbinom(N / 2, 1, en / (p - 1))
  graph   <- matrix(0, p, p)
  graph[upper.tri(graph, diag = FALSE)] <- samples

  # Add directed temporal edges between consecutive waves
  for (w1 in 1:(length(waves) - 1)){
    graph[waves[[w1]], waves[[w1 + 1]]] =
      graph[waves[[w1]], waves[[w1 + 1]]] + diag(length(waves[[1]]))
  }
  graph = graph > 0

  # Latent and/or selection variables Scenarios 1 or 2
  nS = 0
  nL = 0
  #nS = sample(1:2, 1)
  #nL = sample(1:2, 1)


  pL = which(rowSums(graph) >= 2)
  L = sample(pL, min(length(pL), nL))
  actual_indices = setdiff(1:p, L)

  waves_L <- waves
  for (ll in seq_len(length(L))){
    wave_idx = which(sapply(waves_L, FUN=function(X) L[ll] %in% X))
    waves_L[[wave_idx]] = setdiff(waves_L[[wave_idx]], L[ll])
  }

  pS = setdiff(which(colSums(graph) >= 2), L)
```

```r
  S = sample(pS, min(length(pS), nS))
  S_prob = runif(length(S), 0.1, 0.5)


  # Sample number of mixture components and mixing probabilities
  nT     <- sample(3:10, 1)
  T_prob <- runif(nT, 0.3, 0.7)

  # Sample edge weights: Uniform(1, 2) with random signs
  weights <- matrix(
    (runif(p^2) + 1.0) * sample(c(-1, 1), p^2, replace = TRUE),
    p, p
  )
  graph <- graph * weights

  # Partition edges among mixture components
  is   <- which(graph != 0)
  DAGs <- approx_block_random(is, g = length(T_prob))

  return(list(graph   = graph,
              T_prob  = T_prob,
              S_prob  = S_prob,
              waves_L = waves_L,
              waves   = waves,
              S       = S,
              L       = L,
              DAGs    = DAGs))
}

# ------------------------------------------------------------
# Step 2: Sample mixed-type longitudinal observations
# ------------------------------------------------------------

sample_DAGs_mixed <- function(mixDAG, nsamps, binary_prop) {

  nsamps_raw <- nsamps * 8
  p          <- nrow(mixDAG$graph)
  wave_size  = length(mixDAG$waves$w1)
  n_waves    <- length(mixDAG$waves)

  # Assign binary variables
  w1_graph = mixDAG$graph[mixDAG$waves$w1, mixDAG$waves$w1]
  out_degrees <- rowSums(w1_graph != 0)

  n_binary    <- round(wave_size * binary_prop)
  n_cont      <- wave_size - n_binary
  sorted      <- order(out_degrees, decreasing = TRUE)

  continuous_original <- sort(sorted[1:n_cont])
  binary_original     <- sort(sorted[(n_cont + 1):wave_size])

  binary_cols     <- as.integer(sapply(0:(n_waves - 1),
                  function(w) w * wave_size + binary_original))
  continuous_cols <- setdiff(1:p, binary_cols)

  topo_order          <- 1:p
  total_edges         <- sum(mixDAG$graph != 0)
  min_edges_threshold <- max(1, floor(total_edges * 0.3))
```

```r
collected    <- 0
attempts     <- 0
max_attempts <- nsamps_raw * 10
data         <- matrix(0, nsamps_raw, p)

# Generate observations one by one
while (collected < nsamps_raw && attempts < max_attempts) {
  attempts <- attempts + 1

  # Activate mixture components independently
  if (length(mixDAG$T_prob) > 1) {
    Ts <- rbinom(length(mixDAG$T_prob), 1, mixDAG$T_prob)
    Tp <- which(Ts == 0)
    A  <- mixDAG$graph
    for (tp in Tp) A[mixDAG$DAGs[[tp]]] <- 0
  } else {
    A <- mixDAG$graph
  }

  if (sum(A != 0) < min_edges_threshold) next

  # Generate node values in topological order
  x <- numeric(p)
  for (j in topo_order) {
  parents <- which(A[, j] != 0)
  eta <- if (length(parents)>0) sum(A[parents,j]*x[parents]) else 0
    if (j %in% binary_cols) {
      x[j] <- rbinom(1, 1, plogis(eta))
    } else {
      x[j] <- rnorm(1, eta, 1)
    }
  }

  collected         <- collected + 1
  data[collected, ] <- x
}

data <- data[1:collected, , drop = FALSE]

# Apply selection bias if selection variables are present
sample_cut <- c()
for (s in seq_along(mixDAG$S)) {
  if (mixDAG$S[s] %in% continuous_cols) {
    cutoff     <- quantile(data[, mixDAG$S[s]], mixDAG$S_prob[s])
    sample_cut <- c(sample_cut, which(data[, mixDAG$S[s]] <= cutoff))
  }
}
if (length(sample_cut) > 0) data <- data[-sample_cut, ]

n_available <- nrow(data)
if (n_available < nsamps) {
  warning(sprintf("Only %d samples available", n_available))
  nsamps <- n_available
}

data <- data[sample(n_available, nsamps, replace=FALSE), drop=FALSE]
colnames(data) <- 1:p
```

```
  attr(data, "binary_original_vars") <- binary_original
  attr(data, "binary_cols")          <- binary_cols
  attr(data, "continuous_cols")      <- continuous_cols
    return(data)
}
# ------------------------------------------------------------
# Conditional independence test wrapper
# ------------------------------------------------------------

indepTest <- function(x, y, S, suffStat) {
  pval <- mixCItest(x, y, if (length(S) > 0) S else NULL,
                    suffStat = suffStat$data)
  return(pval)
}

# ------------------------------------------------------------
# Step 3: Run experiments
# ------------------------------------------------------------

nsamps      <- 2000
alpha_val   <- 0.01
alg_num     <- 5

res        <- lapply(1:nIter, function(x) vector("list", length = 1))
res_graphs <- vector("list", length = nIter)
suffStat   <- list()

for (t in 1:50) {

  waves1 <- list(w1 = 1:16, w2 = 17:32, w3 = 33:48, w4 = 49:64)
  p      <- 64

  mixDAG <- generate_mix_DAGs(p, 3, waves1)
  synth_data <- sample_DAGs_mixed(mixDAG, nsamps, 0.5)

  res_graphs[[t]] <- mixDAG
  res_graphs[[t]]$binary_original <-attr(synth_data,"binary_original_vars")

  # Randomize variable order to avoid ordering artifacts
  resort_p <- sample(
    c(mixDAG$waves_L$w1, mixDAG$waves_L$w2,
      mixDAG$waves_L$w3, mixDAG$waves_L$w4),
    p - length(mixDAG$L),
    replace = FALSE
  )

  waves <- list(
    w1 = match(mixDAG$waves_L$w1, resort_p),
    w2 = match(mixDAG$waves_L$w2, resort_p),
    w3 = match(mixDAG$waves_L$w3, resort_p),
    w4 = match(mixDAG$waves_L$w4, resort_p)
  )

  suffStat$data <- synth_data[, resort_p]

  wave_names <- c(mixDAG$waves_L$w1, mixDAG$waves_L$w2,
                  mixDAG$waves_L$w3, mixDAG$waves_L$w4)
```

```
  reorder_graph <- function(mat) {
    g <- mat[order(resort_p), order(resort_p)]
    rownames(g) <- colnames(g) <- wave_names
    g
  }

  # Skeleton and IP discovery
  resSkel <- skel_discovery_waves3(suffStat, indepTest,
   alpha = alpha_val, p = ncol(suffStat$data), waves)
  resIP   <- IP_discovery_waves3(suffStat, resSkel$skel, indepTest,
    alpha = alpha_val, p = ncol(suffStat$data), waves)

  # CIM
  st <- proc.time()
  cim_out <- CIM(suffStat, indepTest, alpha = alpha_val,
  p = ncol(suffStat$data), verbose = FALSE, waves = waves,
  prior_know = NULL, resIP = resSkel)
  res[[t]]$cim_waves      <- reorder_graph(cim_out$f_star)
  res[[t]]$cim_waves_time <- proc.time() - st + resSkel$time_skel

  # PC
  st <- proc.time()
  pc_out <- pc_pre(suffStat, indepTest, alpha = alpha_val, p = ncol(suffStat$data),
                   verbose = FALSE, skeleton_pre = resSkel, waves = waves, prior_know
   ↪ = NULL)
  res[[t]]$pc      <- reorder_graph(pc_out$maag)
  res[[t]]$pc_time <- proc.time() - st + resSkel$time_skel

  # FCI
  st <- proc.time()
  fci_out <- fci_pre(suffStat, indepTest, alpha = alpha_val, p = ncol(suffStat$data),
                    verbose = FALSE, skeleton_pre = resIP)
  res[[t]]$fci      <- reorder_graph(fci_out$maag)
  res[[t]]$fci_time <- proc.time() - st + resIP$time_pdsep + resSkel$time_skel

  # RFCI
  st <- proc.time()
  rfci_out <- rfci_pre(suffStat, indepTest, alpha = alpha_val, p =
   ↪ ncol(suffStat$data),
                      verbose = FALSE, skeleton_pre = resSkel)
  res[[t]]$rfci      <- reorder_graph(rfci_out$maag)
  res[[t]]$rfci_time <- proc.time() - st + resSkel$time_skel

  # CCI
  st <- proc.time()
  cci_out <- cci_pre(suffStat, indepTest, alpha = alpha_val, p = ncol(suffStat$data),
                    verbose = FALSE, skeleton_pre = resIP)
  res[[t]]$cci      <- reorder_graph(cci_out$maag)
  res[[t]]$cci_time <- proc.time() - st + resIP$time_pdsep + resSkel$time_skel

  res[[t]]$resIP <- resIP
}


# ------------------------------------------------------------
# Step 4: Compute evaluation metrics
```

```
# ----------------------------------------------------------

metrics_sens    <- matrix(0, 50, alg_num)
metrics_fo      <- matrix(0, 50, alg_num)
metrics_bal_acc <- matrix(0, 50, alg_num)
metrics_OA      <- matrix(0, 50, alg_num)
metrics_time    <- matrix(0, 50, alg_num)

algs_index = c(2, 4, 6, 8, 10)

for (t in 1:nIter){
  for (a in algs_index){

    # compute fallout

    #ground truth
    cum_res2 =
    ↪ res_graphs[[t]]$graph[res_graphs[[t]]$waves_L$w2,res_graphs[[t]]$waves_L$w2]
    #algorithm output
    ans_res2 = res[[t]][[a]][(length(res_graphs[[t]]$waves_L$w1)+1)
    :(length(res_graphs[[t]]$waves_L$w1)+length(res_graphs[[t]]$waves_L$w2)),
    ↪ (length(res_graphs[[t]]$waves_L$w1)+1):(length(res_graphs[[t]]$waves_L$w1)
    +length(res_graphs[[t]]$waves_L$w2))]

    err2 = length(intersect(which(t(cum_res2)!=0),which(t(ans_res2)==3)))

    ###
    cum_res3 =
    ↪ res_graphs[[t]]$graph[res_graphs[[t]]$waves_L$w3,res_graphs[[t]]$waves_L$w3]
    ans_res3 = res[[t]][[a]][(length(res_graphs[[t]]$waves_L$w1)
    +length(res_graphs[[t]]$waves_L$w2)+1):(length(res_graphs[[t]]$waves_L$w1)
    +length(res_graphs[[t]]$waves_L$w2)+length(res_graphs[[t]]$waves_L$w3)),
     (length(res_graphs[[t]]$waves_L$w1)+length(res_graphs[[t]]$waves_L$w2)+1):
    ↪ (length(res_graphs[[t]]$waves_L$w1)+length(res_graphs[[t]]$waves_L$w2)
     +length(res_graphs[[t]]$waves_L$w3))]
    err3 = length(intersect(which(t(cum_res3)!=0),which(t(ans_res3)==3)))


    ###
    cum_res4 =
    ↪ res_graphs[[t]]$graph[res_graphs[[t]]$waves_L$w4,res_graphs[[t]]$waves_L$w4]
    ans_res4 =res[[t]][[a]][(length(res_graphs[[t]]$waves_L$w1)
    +length(res_graphs[[t]]$waves_L$w2)+length(res_graphs[[t]]$waves_L$w3)+1):
    ↪ (length(res_graphs[[t]]$waves_L$w1)+length(res_graphs[[t]]$waves_L$w2)+
    length(res_graphs[[t]]$waves_L$w3)+length(res_graphs[[t]]$waves_L$w4)),
    (length(res_graphs[[t]]$waves_L$w1)+length(res_graphs[[t]]$waves_L$w2)
    +length(res_graphs[[t]]$waves_L$w3)+1):(length(res_graphs[[t]]$waves_L$w1)
    +length(res_graphs[[t]]$waves_L$w2)+length(res_graphs[[t]]$waves_L$w3)
    +length(res_graphs[[t]]$waves_L$w4))]
    err4 = length(intersect(which(t(cum_res4)!=0),which(t(ans_res4)==3)))

    cres2 = sum(t(cum_res2)!=0)
    cres3 = sum(t(cum_res3)!=0)
    cres4 = sum(t(cum_res4)!=0)

    if ((cres2+cres3+cres4) > 0){
      errF = (err2+err3+err4)/(cres2+cres3+cres4)
    } else{
```

```
  errF = 0;
}
metrics_fo[t,which(algs_index==a)]=errF # record fallout


# compute sensitivity
cum_res2 =
↪ res_graphs[[t]]$graph[res_graphs[[t]]$waves_L$w2,res_graphs[[t]]$waves_L$w2]
ans_res2 = res[[t]][[a]][(length(res_graphs[[t]]$waves_L$w1)+1):
(length(res_graphs[[t]]$waves_L$w1)+length(res_graphs[[t]]$waves_L$w2)),
 (length(res_graphs[[t]]$waves_L$w1)+1):(length(res_graphs[[t]]$waves_L$w1)
 +length(res_graphs[[t]]$waves_L$w2))]
err2 = length(intersect(which(cum_res2!=0),which(t(ans_res2)==3)))

cum_res3 =
↪ res_graphs[[t]]$graph[res_graphs[[t]]$waves_L$w3,res_graphs[[t]]$waves_L$w3]
ans_res3 = res[[t]][[a]][(length(res_graphs[[t]]$waves_L$w1)
+length(res_graphs[[t]]$waves_L$w2)+1):(length(res_graphs[[t]]$waves_L$w1)
+length(res_graphs[[t]]$waves_L$w2)+length(res_graphs[[t]]$waves_L$w3)),
 (length(res_graphs[[t]]$waves_L$w1)+length(res_graphs[[t]]$waves_L$w2)+1):
 (length(res_graphs[[t]]$waves_L$w1)+length(res_graphs[[t]]$waves_L$w2)
 +length(res_graphs[[t]]$waves_L$w3))]
err3 = length(intersect(which(cum_res3!=0),which(t(ans_res3)==3)))

###
cum_res4 =
↪ res_graphs[[t]]$graph[res_graphs[[t]]$waves_L$w4,res_graphs[[t]]$waves_L$w4]
ans_res4 = res[[t]][[a]][(length(res_graphs[[t]]$waves_L$w1)
+length(res_graphs[[t]]$waves_L$w2)+length(res_graphs[[t]]$waves_L$w3)+1):
(length(res_graphs[[t]]$waves_L$w1)+length(res_graphs[[t]]$waves_L$w2)
+length(res_graphs[[t]]$waves_L$w3)+length(res_graphs[[t]]$waves_L$w4)),
(length(res_graphs[[t]]$waves_L$w1)+length(res_graphs[[t]]$waves_L$w2)
+length(res_graphs[[t]]$waves_L$w3)+1):(length(res_graphs[[t]]$waves_L$w1)
+length(res_graphs[[t]]$waves_L$w2)+length(res_graphs[[t]]$waves_L$w3)
+length(res_graphs[[t]]$waves_L$w4))]
err4 = length(intersect(which(cum_res4!=0),which(t(ans_res4)==3)))

cres2 = sum(cum_res2!=0)
cres3 = sum(cum_res3!=0)
cres4 = sum(cum_res4!=0)
if ((cres2+cres3+cres4) > 0){
  err = (err2+err3+err4)/(cres2+cres3+cres4)
} else{
  err = 0;
}
# record sensitivty
metrics_sens[t,which(algs_index==a)]=err

# record overall score
metrics_OA[t,which(algs_index==a)]=sqrt( metrics_fo[t,which(algs_index==a)]^2 +
↪ (1-metrics_sens[t,which(algs_index==a)])^2 )

# record balanced accuracy
metrics_bal_acc[t, which(algs_index==a)] = (
  metrics_sens[t, which(algs_index==a)] +
    (1 - metrics_fo[t, which(algs_index==a)])
) / 2
```

```
    # record time
    metrics_time[t,which(algs_index==a)]=res[[t]][[a+1]][3]
  }
}


# ------------------------------------------------------------
# Step 5: Report results
# ------------------------------------------------------------

boxplot(metrics_sens)
boxplot(metrics_fo)
boxplot(metrics_bal_acc)
boxplot(metrics_OA)
boxplot(metrics_time)
```
